# A Tiered 3D CNN Framework for Forensic Sex Estimation from CBCT: Matched Comparison of Full-Skull Volumes and Mandible-Only Masks

**DOI:** 10.3390/biology15201891

**Published:** 2026-10-20

**Authors:** Yubo Lei, Xiaocheng Yang, Shuanglong Feng, Songrui Yang, Shaoxiang Liu, Shuhua Ning, Xiaohe Pu, Qingshan Wang

**Affiliations:** 1 College of Mechanical and Vehicle Engineering, Chongqing University, Chongqing 400030, China; 20230701075g@stu.cqu.edu.cn; 2 Chongqing Institute of Green and Intelligent Technology, Chinese Academy of Sciences, Chongqing 400714, China; fengshuanglong@cigit.ac.cn (S.F.); liushaoxiang@cigit.ac.cn (S.L.); 3 Chongqing Public Security Bureau, Chongqing 401147, China; xql16681@163.com (X.Y.); yxl0369@163.com (X.P.); 4 Key Laboratory of Functional Composite Materials of Guizhou Province, College of Big Data and Information Engineering, Guizhou University, Guiyang 550025, China; yang_sr1993@163.com; 5 Chongqing Police College, Chongqing 401331, China; shuhuaning2025@163.com

**Keywords:** forensic sex estimation, mandible, cone-beam computed tomography, 3D convolutional neural network, matched evaluation

## Abstract

Deep learning has advanced automated sex estimation from craniofacial imaging, but differences in cohorts, input representations, and protocols between studies leave the performance gap between full-skull and mandible-only inputs insufficiently quantified. We compared the two inputs under matched conditions using cone-beam computed tomography (CBCT) scans of 353 Chinese Han individuals (102 males and 251 females, aged 6–78 years). From each scan, we derived a full-skull grayscale volume ($128^{3}$) and an automatically segmented mandibular mask ($80^{3}$). The same lightweight three-dimensional (3D) convolutional neural network (about 572,000 parameters) was trained on each input with identical five-fold cross-validation splits and training protocol. Balanced accuracy (BAcc) was 96.20% ± 3.66% for the full-skull input and 88.67% ± 4.58% for the mandible-only input (paired difference in pooled BAcc, 7.52 percentage points, 95% confidence interval (CI) 4.55–10.50). Mandible-only BAcc was 93.17% ± 2.63% at 19–50 years (n=259), whereas pooled BAcc was 66.67% at ≤17 years and 79.80% above 50 years. In this single-center cohort, the full-skull input performed better overall, and the mandible-only input achieved high accuracy only in adults aged 19–50 years. Applying these subgroup results requires an independent age assessment, and external validation is needed before forensic use.

## Simple Summary

Deep learning enables automated sex estimation from skull images for forensic identification. Differences in study populations and methods have obscured the performance gap between full-skull and lower-jaw inputs. We compared these inputs using three-dimensional scans from 353 Han Chinese individuals. Separate models were trained using the same network design, training procedure, and cross-validation splits. Performance was measured by balanced accuracy, which averages the correct classification rates for males and females. Mean balanced accuracy reached 96.2% with full-skull inputs and 88.7% with lower-jaw inputs. The lower-jaw model achieved 93.2% among adults aged 19 to 50 years, with lower performance in children and older individuals. Within the lower-jaw region, shape-based representations outperformed images that also retained variations in brightness. Within this cohort, the matched comparisons showed higher balanced accuracy with full-skull inputs and age-associated differences in lower-jaw performance. The findings guide anatomical input selection and the interpretation of deep learning results in forensic sex estimation.

## 1. Introduction

Deep learning has facilitated advances in forensic sex estimation, a fundamental task in forensic anthropology that helps narrow the search space for human identification [1,2,3]. Although the pelvis is generally regarded as the most sexually dimorphic skeletal region, it may be unavailable in fragmented or incomplete remains. Under such circumstances, the cranium and mandible provide well-documented sexually dimorphic features [4,5]. In particular, the mandible is one of the most durable craniofacial bones and often survives when other craniofacial structures do not [6]. A key practical question is how much sex-estimation performance is lost when the available evidence is reduced from the full skull to the mandible alone.

### 1.1. Mandibular Evidence in Sex Estimation

The mandible is an established source of sexually dimorphic skeletal information. Traditional studies converted this information into manually defined linear, angular, or morphoscopic variables and analyzed them with discriminant function analysis (DFA). Within single populations, such measurements typically reach high-70s to high-80s accuracy [7,8,9]. Despite these within-population results, metric and morphoscopic approaches remain dependent on operator-defined features and often transfer poorly across populations. An early study reported up to 99% accuracy for mandibular ramus flexure [10]. However, subsequent blind tests reported substantially lower accuracies of 62.5% and 79.1% [11,12]. Discriminant functions developed in one population also perform poorly in another [7], in line with meta-analytic evidence of substantial between-population heterogeneity in mandibular measurements [4].

The move to three-dimensional imaging addressed part of this problem. Mandibular morphology could now be measured on computed tomography (CT) or cone-beam computed tomography (CBCT) volumes rather than on dry bones or two-dimensional projections. However, most CT and CBCT studies still extracted predefined anatomical measurements first and then performed classification. A CT measurement study reported about 90% correct classification for complete mandibles [13], with broadly similar performance reported for Chinese Han CBCT cohorts [14,15] and other populations [16,17,18]. Geometric morphometric analyses extended this approach with size, shape, and dense semi-landmark schemes [6,19,20], but remained dependent on operator-defined landmarks. The interaction between age-related mandibular changes and sex differences has also been documented [21]. Across both metric and morphometric work, the dependence on operator-extracted features remained the principal bottleneck for automation, motivating the shift toward learned representations.

Recent deep-learning studies have reduced this dependence on hand-crafted features. Two-dimensional convolutional neural networks on cephalometric radiographs and cranial photographs can reach high accuracy [22,23]. However, performance is sensitive to the imaging view. Sex-specific classification accuracy can be markedly asymmetric, reaching 100% for males and 76.9% for females in one photographed-mandible deep-learning (DL) study [24]. Three-dimensional deep learning on mandibular evidence has so far been pursued mainly on point-cloud or projection representations rather than on segmented voxel volumes. An adapted PointNet++ trained on Croatian mandibular point clouds reached 93%/92% cross-validation/test accuracy [25], with saliency concentrated on the chin, gonial angle, and condylar region. A CT-based mandibular deep-learning study reported 92.50% accuracy with a hybrid model [26]. The absence of segmented voxel-volume approaches motivates the binary-mask Tier 2 path introduced here. Selected shallow machine-learning baselines on mandibular measurements [27,28] are listed in Supplementary Table S1.

The mandible therefore offers an informative but localized source of sex-related skeletal information, with predictive performance shaped by how this evidence is represented in the model input.

### 1.2. Skull-Based Deep Learning for Sex Estimation

Skull-level inputs have also been used for deep-learning sex estimation, with reported accuracies in a broadly similar range to mandibular-only studies. A convolutional neural network (CNN) approach using adult skull CT images reached 95% accuracy [2]. Deep feature selection and fusion on Turkish skull CT reached 96.4% [3]. A multi-task model on CBCT-derived panoramic slices, using a convolutional block attention module (CBAM), reached 95.3% sex-classification accuracy [29]. For three-dimensional (3D) skull representations, ResNet3D, PointNet++, and MeshNet were compared, with PointNet++ achieving the strongest area under the curve (AUC)-based performance among the three architectures [30]. Work on related skeletal-imaging tasks under comparable data constraints is also relevant. Deep-learning approaches to bone age assessment have been developed for limited single-center datasets [31,32], a constraint shared by forensic cohorts.

The limitation is that these studies were conducted in different cohorts (Australian, Turkish, mixed European) and differ methodologically from mandibular work. These skull-level studies provide important context, but their cohorts, input representations, and evaluation protocols differ from those used in mandibular studies.

### 1.3. Need for Same-Cohort Input Comparison

Existing mandibular and skull-level studies cannot be compared by simply subtracting their reported accuracies, because they differ in study population, imaging and input representation, and modeling and evaluation design. For this reason, a same-cohort design is required, in which the same cases are evaluated under identical fold assignments, a shared backbone, and a common training protocol. This removes the major architecture- and protocol-related sources of variation. The remaining contrast is the performance gap between the two predefined input paths under a shared modeling setup.

Evaluating both inputs within one cohort removes most of this ambiguity. The two inputs still differ in more than the region they cover, so the gap should not be read as the effect of anatomical scope alone.

We implement two parallel input pathways. The full-skull pathway (Tier 1) processes grayscale CBCT volumes of size $128^{3}$, whereas the mandible-only pathway (Tier 2) uses binary segmentation masks of size $80^{3}$. Both pathways employ the same backbone architecture and stratified five-fold data split. The lightweight backbone, comprising 572,338 parameters, reduces the risk of overfitting in a modest and imbalanced cohort while maintaining identical model capacity across the two input types.

To the best of our knowledge, a same-cohort, protocol-matched 3D CNN evaluation directly comparing full-skull grayscale CBCT volumes with mandible-only binary masks for forensic sex estimation has not been reported. We address this gap in a Chinese Han cohort through the following three contributions.

(1) We quantify the full-skull advantage over mandible-only input using the same cases, backbone architecture, fold assignment, and training protocol, with case-level paired bootstrap confidence intervals and paired McNemar testing.
(2) A four-configuration ablation separates anatomical scope, input modality, and spatial resolution, showing that anatomical scope is the primary contributor and that, once the scope is restricted to the mandible, binary masks outperform mandibular grayscale input in this cohort.
(3) We characterize mandible-only performance across pre-specified age strata, reporting the 19–50 adult subgroup as a descriptive performance analysis rather than an age-conditioned inference rule.

We hypothesized that, with cases, backbone, cross-validation folds, and training protocol matched, the full-skull input would achieve higher balanced accuracy (BAcc) than the mandible-only input. Contributions (2) and (3) address secondary aims.

## 2. Materials and Methods

**Figure 1:**
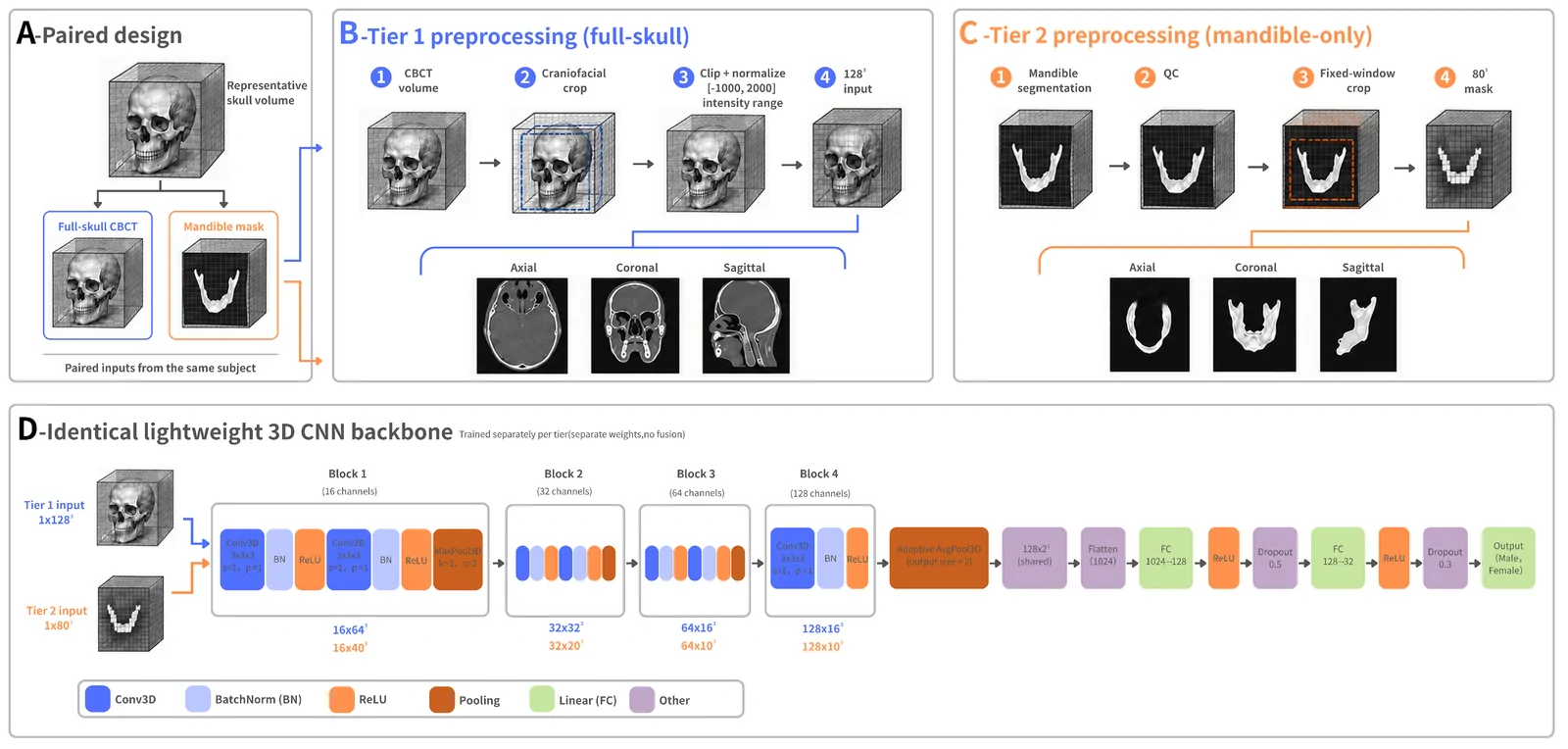
Overview of the tiered three-dimensional (3D) framework. (**A**) Paired full-skull cone-beam computed tomography (CBCT) volume and mandibular mask from each scan in native coordinates, without registration. (**B**) Tier 1 preprocessing to a 128^3^ full-skull grayscale input. (**C**) Tier 2 preprocessing to an 80^3^ mandibular binary mask. (**D**) Identical lightweight 3D convolutional neural network (CNN) backbone with separate training and weights per tier, without fusion. QC, quality control; Conv3D, 3D convolution; BN, batch normalization; ReLU, rectified linear unit; FC, fully connected.

The experimental framework consisted of four stages, as illustrated in Figure 1. Section 2.1 describes data curation and quality control, while Section 2.2 details the tier-specific preprocessing of full-skull and mandibular inputs. Section 2.3 and Section 2.4 present the lightweight 3D CNN and its training under a unified plateau-scheduled protocol, respectively. The tiered evaluation strategy is described in Section 2.5, and the morphometric baseline models used for comparison are introduced in Section 2.6.

### 2.1. Study Cohort

This study included a curated cohort of 353 paired craniofacial imaging cases from a Chinese Han population. The cohort consisted of 102 male and 251 female cases ranging in age from 6 to 78 years, with a median age of 22 years and an interquartile range (IQR) of 20–35 years. Each case provided a native full-skull CBCT volume and an automatically segmented mandibular mask from the same Digital Imaging and Communications in Medicine (DICOM) series. The study size was based on the eligible CBCT cases obtained through the project-coordinated collection. Sex was not used as a case-selection criterion, and the female predominance reflects the composition of the available source data.

The dataset was curated and analyzed at the Chongqing Institute of Green and Intelligent Technology, Chinese Academy of Sciences. It was obtained from de-identified clinical maxillofacial CBCT records of Chinese Han subjects. All cases were acquired on a single LargeV Smart3DX CBCT system (LargeV Instrument Corp., Ltd., Beijing, China). Additional acquisition-parameter audit details are provided in Supplementary Table S3.

The two tiers used different source volumes from the same acquisition. For Tier 1, the native DICOM volume was retained for grayscale preprocessing. For Tier 2, mandibular masks were generated automatically using an in-house nnU-Net v2 segmentation model [33,34,35], trained on 384 craniofacial CT volumes to segment seven head-and-neck structures. Only the mandibular label was retained as a binary volumetric mask, while the remaining labels were discarded. On its internal held-out validation split (fold 0 of the 384-case craniofacial CT training set), the segmentation model reached a mandible-class Dice of 0.96. Target-domain segmentation accuracy on the dental-CBCT classification cohort was not separately quantified. Mask quality on the present cohort was instead controlled by the connected-component quality control described in Section 2.1.2, which excluded 6 of 359 masks. Inference used the three-dimensional full-resolution configuration, with a target voxel spacing of 0.5 mm isotropic and CT intensity normalization. The resulting masks were mapped back to the native 0.25 mm acquisition grid (Section 2.1.2). Segmentation was run within 3D Slicer (v5.10.0).

Each mask corresponded spatially to its source DICOM series in the native coordinate space, and no cross-subject registration or template alignment was applied. Throughout this study, ‘mandible-only’ denotes the automatically segmented mandibular representation, including the alveolar process. Only the mandible label was retained. The separately labeled lower and upper teeth were not merged into Tier 2. Tier 2 used the resampled occupancy mask of this label (Section 2.2), without CBCT intensities. Because the segmentation model was trained on craniofacial CT and applied to dental CBCT, differences in imaging modality and field of view occasionally produced fragmented masks. These were removed by the geometric quality-control rule described in Section 2.1.2.

Sex and age metadata were obtained from clinical records and linked to each case through structured metadata tables. Sex labels were not inferred from CBCT imaging features and were cross-checked against these records by our team. Individual masks were not manually corrected as a routine step. Instead, automated segmentation outputs underwent case-level quality control, as described in Section 2.1.2. Each subject contributed a single CBCT acquisition and appeared in only one cross-validation fold.

The inclusion, exclusion, and quality-control logic are detailed in Section 2.1.1 and Section 2.1.2. Table 1 summarizes the analytic cohort composition, age distribution, and cross-validation setup.

#### 2.1.1. Inclusion and Exclusion Criteria

Cases were included if they had (1) a valid mandibular segmentation, (2) linked sex and age metadata, and (3) a mandibular mask that passed the connected-component quality control detailed in Section 2.1.2.

#### 2.1.2. Data Curation and Quality Control

Each mandibular mask underwent automated quality control before model training. A mask was excluded if it was empty, over-segmented to include non-mandibular structures, or fragmented into disconnected components. Fragmentation was identified by connected-component analysis, rejecting any mask whose largest component held less than 95% of foreground voxels while its second-largest exceeded 50,000 voxels. Of the 359 segmented masks, six were excluded during quality control, leaving a final cohort of 353 cases.

The source CBCT acquisitions, all obtained at 100 kVp, used isotropic voxel spacing of 0.250 mm (n=337), 0.125 mm (n=7), or 0.088 mm (n=9). The 16 higher-resolution acquisitions were resampled to a common 0.25 mm isotropic grid during mask generation. CBCT voxel intensities are not calibrated to true Hounsfield units on the medical-CT scale. Intensity clipping in Tier 1 preprocessing (Section 2.2) therefore operates on the vendor-reported nominal intensity scale, not on calibrated Hounsfield units. A full acquisition audit is provided in Supplementary Table S3.

### 2.2. Preprocessing

Each tier used a dedicated preprocessing routine that mapped its source volume to a normalized fixed-size input, as summarized in Figure 1. Tier 1 operated on full-skull grayscale CBCT volumes, and Tier 2 on mandibular binary masks.

For Tier 1, the native DICOM volume of each case was read on the vendor-reported nominal intensity scale. A bounding box was computed from voxels exceeding a nominal intensity of 300, an empirical threshold that approximately separates mineralized bone from soft tissue and background on the LargeV Smart3DX scale. The threshold defined the bounding box only. The cropped grayscale volume retained the dentition and soft tissue within that box, where present. The same threshold was used for all 353 cases across the five folds, without fold-specific re-optimization. Voxel intensities were clipped to [−1000,2000] on the same scale to standardize the dynamic range. Each cropped volume was subsequently resampled to a fixed 128×128×128 grid using trilinear interpolation and min–max normalized to [0,1].

For Tier 2, the corresponding mandibular masks were loaded in Neuroimaging Informatics Technology Initiative (NIfTI) format. A fixed window of 576×460×372 voxels was centered on the foreground centroid, where the foreground was defined as voxels with values greater than 0.5. Zero padding was introduced whenever the window extended beyond the image boundaries. This fixed-window approach standardized the spatial context surrounding the mandible across cases while preserving its local morphology. The cropped binary segmentation was resampled to 80×80×80 voxels by trilinear interpolation, yielding an occupancy mask with interior voxels equal to 1, background voxels equal to 0, and surface voxels taking partial-volume values in [0,1]. Throughout this paper, “binary mask” refers to this resampled occupancy representation derived from the binary source segmentation. This representation retains mandibular geometry while discarding CBCT intensity. The volume was then min–max normalized to [0,1].

The two preprocessing routines generated inputs with different scales and modalities, consistent with their distinct anatomical contexts. Tier 1 produced a $128^{3}$ grayscale CBCT volume, whereas Tier 2 produced an $80^{3}$ binary-mask volume.

The two intermediate configurations examined in Section 3.6 were designed to differ from their respective reference tiers along only one axis. Tier 1-80 followed the same full-skull grayscale preprocessing routine as Tier 1, including the intensity-300 bounding box, [−1000,2000] intensity clipping, and per-volume min–max normalization. However, the volume was resampled to $80^{3}$ instead of $128^{3}$, thereby isolating the effect of spatial resolution. Tier 2-Gray followed the same mandibular preprocessing routine as Tier 2, including the foreground-centroid window center, fixed 576×460×372 crop, $80^{3}$ trilinear resampling, and per-volume min–max normalization, but differed in input modality alone. Voxels within the mandibular mask retained the grayscale intensities from the underlying CBCT volume in native space. These intensities were clipped to [−1000,2000] and min–max normalized as in Tier 1, whereas voxels outside the mask were assigned a value of zero. Thus, each ablation configuration kept all preprocessing steps unchanged except for the single factor being evaluated.

During model training, spatial augmentation was restricted to the training split. The applied transformations included random flipping along each axis, random rotations within ±10 degrees, and random translations of up to ±4 voxels along each axis. Random isotropic scaling between 0.92 and 1.08 and additive Gaussian noise with a sigma of 0.015 completed the set. These parameters were identical for both tiers, and augmentation was disabled during validation and inference.

### 2.3. Lightweight 3D CNN Backbone

Both tiers used the same lightweight 3D CNN backbone. The network stacked four 3D convolutional blocks with increasing channel depth (16, 32, 64, and 128). Each block used 3×3×3 kernels with stride 1 and padding 1, followed by batch normalization [36] and a rectified linear unit. Blocks 1 to 3 each held two convolutional layers and a max-pooling layer (kernel 2, stride 2) for spatial downsampling. Block 4 held a single convolutional layer followed by adaptive average pooling to 2×2×2. An optional squeeze-and-excitation (SE) channel-attention module [37] with reduction ratio 4 could be inserted after the convolutional layers of each block. The classifier head stacked three fully connected layers (1024 to 128 to 32 to 2) with rectified-linear activation and dropout rates of 0.5 and 0.3.

The SE-enabled backbone held 583,218 trainable parameters, and the SE-disabled variant held 572,338. Both are well below the tens of millions of parameters typical of standard 3D ResNet variants [38], which suits the modest size of the present cohort. This lightweight design balances representational capacity against optimization stability under a small, class-imbalanced forensic dataset, with the full layer specification given in Table 2. A larger ResNet3D-18 backbone was additionally evaluated as a contextual capacity reference and is reported in Supplementary Note S3.

To assess the contribution of the SE modules, both variants were trained and evaluated under identical conditions, with the ablation reported in Supplementary Note S2.

### 2.4. Training Protocol

Five-fold stratified cross-validation was used throughout internal development to preserve class proportions across folds. Folds were generated by shuffled stratified splitting under a fixed seed of 42. The same fold assignment was used for Tier 1 and Tier 2, so that case-level paired comparisons between tiers remain valid.

For Tier 2, training used a batch size of 4 with gradient accumulation over 4 steps, giving an effective batch size of 16. Tier 1 instead used a batch size of 2 with accumulation over 8 steps to fit the larger input in graphics processing unit (GPU) memory. Both configurations therefore shared an effective batch size of 16.

Optimization used AdamW [39] with an initial learning rate of $5\times 10^{-4}$ and weight decay of $1\times 10^{-3}$. The schedule combined a 5-epoch linear warmup with a subsequent ReduceLROnPlateau policy (factor 0.5, patience 15, minimum rate $1\times 10^{-6}$) over at most 300 epochs. Training stopped early after 100 consecutive epochs without improvement in validation balanced accuracy.

Within each outer fold, a stratified inner validation split (20% of the training cases, seed 42) was held out, and the best checkpoint was chosen by validation balanced accuracy. The same inner-split protocol was applied to all deep-learning models and to the morphometric multilayer perceptron (MLP) baseline for comparable model selection. No probability calibration [40] was applied, and all reported probabilities are raw softmax outputs.

Class imbalance was handled at two levels, namely class-weighted cross-entropy with label smoothing at the loss level and a weighted random sampler at the data level. Class weights followed the balanced convention defined as

(1) $$w_{c}=\frac{N_{\mathrm{total}}}{2\cdot N_{c}},\;c\in \left\{\mathrm{male},\mathrm{female}\right\},$$

where $N_{\mathrm{total}}$ is the number of training-set cases in the current fold and $N_{c}$ is the number of training-set cases of class *c*.

The weighted random sampler drew each training sample *i* with probability proportional to

(2) $$p_{i}\propto w_{i}=\frac{1}{\sqrt{N_{c(i)}}},\;i=1,\ldots ,N_{\mathrm{train}},$$

where c(i) denotes the class of sample *i* and $N_{c(i)}$ is the number of training-set cases of that class. The square-root scaling provides a milder rebalancing than full inverse-frequency sampling.

The training objective combined the class-weighted cross-entropy with label smoothing as

(3) $$L_{\mathrm{LS}}=-\sum_{c=1}^{C}w_{c}\cdot {\tilde{y}}_{c}\cdot \log\left({\hat{p}}_{c}\right),$$

(4) $${\tilde{y}}_{c}=\left(1-\varepsilon \right)\cdot 1\left[c=y\right]+\frac{\varepsilon }{C},$$

where C=2 is the number of classes, y∈{male,female} is the ground-truth label, ${\hat{p}}_{c}$ is the softmax-predicted probability of class *c*, $w_{c}$ is the class weight from Equation (1), 1[·] is the indicator function, and the smoothing parameter was fixed at ε=0.1.

The same optimization and schedule were applied to both Tier 1 and Tier 2 training runs without tier-specific hyperparameter tuning.

### 2.5. Tiered Evaluation

Evaluation proceeded directly from each tier’s out-of-fold (OOF) predictions, with no post-hoc fusion step. For each outer fold, the inner-split checkpoint was applied to the held-out fold without retraining, so no inner-validation case entered its own evaluation. Tier 1 and Tier 2 metrics were each computed on the OOF predictions across all 353 cases. An adult subgroup report was additionally derived from the Tier 2 OOF predictions, restricted to the 19–50 year window (n=259).

This window was defined a priori from the forensic anthropology literature [21,41] as the prime adult casework demographic. It is bounded below by the end of subadult craniofacial development and above by the onset of age-related mandibular resorption, and was not derived from the predictions themselves.

No ensemble, threshold tuning, age-dependent decision rule, or demographic-conditioned selection was used in the primary analysis, and the male-class decision threshold was held at the default 0.5 throughout.

In the primary CNN analysis, chronological age was not supplied as an explicit input. The 19–50-year results are subgroup estimates from the all-age models. To examine age jointly with input type, we performed post-hoc analyses of the fixed out-of-fold predictions. Case-level misclassification was modeled with a generalized estimating equation (binomial family, logit link, exchangeable working correlation within cases, robust standard errors). Covariates were input tier, age group (≤18, 19–50, and >50 years), and sex. The ≤17 and 18-year strata were reported separately in the descriptive analyses but combined here because of their small size. A tier × age-group interaction was tested with a Wald test. For each tier, misclassification was also modeled by logistic regression on age group and sex. In addition, we evaluated an age-only classifier (logistic regression on a cubic spline of age, trained on the same outer folds) and computed age-standardized BAcc by weighting male and female recall in the five age strata by the whole-cohort stratum proportions (Supplementary Table S10).

A sensitivity analysis examined the effect of including the lower teeth in the Tier 2 input. For each case, the corresponding automatically segmented lower-teeth label was added to the mandible label within the original mandibular crop. The combined mask then underwent the original resampling and normalization pipeline. Tier 2 was retrained on this input with the original case-level fold assignments, seeds, and training protocol. Its out-of-fold predictions were compared with the saved predictions of the original input. The paired difference in pooled BAcc was estimated from 5000 sex-stratified case-paired bootstrap resamples (seed 42, percentile intervals, Supplementary Table S11).

Metrics are reported as fold-mean ± standard deviation (SD) of per-fold BAcc. Pooled cohort-level metrics computed over all OOF predictions serve as a sanity check, and are reported in Section 3 only when they differ materially from the fold-mean summaries.

An exploratory age-stratified fusion of the whole-volume and fixed-window mandibular branches was evaluated during development. It did not improve over the retained tiered framework and was not kept. Full numerical results appear in Supplementary Note S1.

The complete procedure, including the paired-bootstrap and McNemar tests reported in Section 3, is summarized in Algorithm 1.
**Algorithm 1** Tiered out-of-fold evaluation pipeline**Input:**Cases $C={\left\{\left(x_{i}^{\left(1\right)},x_{i}^{\left(2\right)},y_{i},a_{i}\right)\right\}}_{i=1}^{N}$, N=353        $x_{i}^{\left(1\right)}$: Tier 1 grayscale CBCT volume ($128^{3}$); $x_{i}^{\left(2\right)}$: Tier 2 mandibular binary mask ($80^{3}$)        $y_{i}\in \left\{M,F\right\}$: sex label; $a_{i}\in R$: age (years)Stratified 5-fold split $F=\{F_{1},\ldots ,F_{5}\}$, seed =42Backbone $f_{\theta }$ (572,338-param 3D CNN; Section 2.3)Loss $L_{\mathrm{LS}}$ (Equation (3)); class weights $w_{c}$ (Equation (1)); sampler $w_{i}$ (Equation (2)); schedule *T* (Section 2.4)Adult window $A=\{i:19\leq a_{i}\leq 50\}$**Output:**OOF prediction tables $P^{\left(1\right)},P^{\left(2\right)}$Fold-mean BAcc $\mu_{1},\mu_{2},\mu_{2,\mathrm{adult}}$ and SD $\sigma_{1},\sigma_{2},\sigma_{2,\mathrm{adult}}$Paired-bootstrap 95% confidence interval (CI) for $\Delta \mathrm{BAcc}={\mathrm{BAcc}}_{1}-{\mathrm{BAcc}}_{2}$Exact McNemar *p*-value $p_{\mathrm{McN}}$ for paired prediction disagreement1:**for** k=1 to 5 **do**2:      $I_{\mathrm{train}}\leftarrow \left\{i:i\notin F_{k}\right\}$3:      $I_{\mathrm{val}}\leftarrow \mathrm{StratifiedSplit}\left(I_{\mathrm{train}},\mathrm{ratio}=0.2,\mathrm{seed}=42\right)$4:      **for** t∈{1,2} **do**5:            $\theta_{t,k}^{*}\leftarrow \mathrm{Train}(f_{\theta };\left\{\left(x_{i}^{\left(t\right)},y_{i}\right):i\in I_{\mathrm{train}}\setminus I_{\mathrm{val}}\right\},$                        $\mathrm{val}=\left\{\left(x_{i}^{\left(t\right)},y_{i}\right):i\in I_{\mathrm{val}}\right\},L_{\mathrm{LS}},w_{c},w_{i},T)$6:            **for** $i\in F_{k}$ **do**7:                 $P^{\left(t\right)}\left[i\right]\leftarrow \mathrm{softmax}\left(f_{\theta_{t,k}^{*}}\left(x_{i}^{\left(t\right)}\right)\right)$8:            **end for**9:      **end for**10:    **for** t∈{1,2} **do**11:          ${\mathrm{BAcc}}_{t,k}\leftarrow \mathrm{BalancedAccuracy}\left(\{\left(\arg\max P^{\left(t\right)}\left[i\right],y_{i}\right):i\in F_{k}\}\right)$12:    **end for**13:    ${\mathrm{BAcc}}_{2,k,\mathrm{adult}}\leftarrow \mathrm{BalancedAccuracy}\left(\{\left(\arg\max P^{\left(2\right)}\left[i\right],y_{i}\right):i\in F_{k}\cap A\}\right)$14:**end for**15:$\mu_{1}\leftarrow {\mathrm{mean}}_{k}\left({\mathrm{BAcc}}_{1,k}\right)$, $\sigma_{1}\leftarrow {\mathrm{std}}_{k}\left({\mathrm{BAcc}}_{1,k}\right)$16:$\mu_{2}\leftarrow {\mathrm{mean}}_{k}\left({\mathrm{BAcc}}_{2,k}\right)$, $\sigma_{2}\leftarrow {\mathrm{std}}_{k}\left({\mathrm{BAcc}}_{2,k}\right)$17:$\mu_{2,\mathrm{adult}}\leftarrow {\mathrm{mean}}_{k}\left({\mathrm{BAcc}}_{2,k,\mathrm{adult}}\right)$, $\sigma_{2,\mathrm{adult}}\leftarrow {\mathrm{std}}_{k}\left({\mathrm{BAcc}}_{2,k,\mathrm{adult}}\right)$18:$\left[\Delta {\mathrm{BAcc}}_{\mathrm{lo}},\Delta {\mathrm{BAcc}}_{\mathrm{hi}}\right]\leftarrow \mathrm{StratifiedPairedBootstrap}\left(P^{\left(1\right)},P^{\left(2\right)},\left\{y_{i}\right\};B=5000,\mathrm{strata}=\left\{y_{i}\right\}\right)$19:$p_{\mathrm{McN}}\leftarrow \mathrm{McNemarExactTest}\left(P^{\left(1\right)},P^{\left(2\right)},\left\{y_{i}\right\}\right)$20:**return** $P^{\left(1\right)},P^{\left(2\right)},\left(\mu_{1},\sigma_{1}\right),\left(\mu_{2},\sigma_{2}\right),\left(\mu_{2,\mathrm{adult}},\sigma_{2,\mathrm{adult}}\right),$         $\left[\Delta {\mathrm{BAcc}}_{\mathrm{lo}},\Delta {\mathrm{BAcc}}_{\mathrm{hi}}\right],p_{\mathrm{McN}}$

### 2.6. Baseline Models and Feature Construction

To contextualize the proposed backbone, four morphometric machine-learning baselines were evaluated, namely logistic regression, random forest, a multilayer perceptron, and a support vector machine with a radial-basis-function kernel. All four were trained under the same five-fold stratified cross-validation folds (seed 42) on the final 353-case cohort as the deep-learning models.

For each mandibular mask, seven shape descriptors were computed from the foreground by connected-component analysis. The seven descriptors are listed below.

(1) Total foreground volume, as a voxel count.
(2) Bounding-box anterior–posterior extent.
(3) Bounding-box medio-lateral extent.
(4) Bounding-box supero-inferior extent.
(5) Elongation, the ratio of maximum to minimum bounding-box extent.
(6) Surface area, from marching-cubes triangulation.
(7) Solidity, foreground volume divided by convex-hull volume.

These seven features were fixed a priori by morphometric convention and were not selected from classification performance. The feature set was z-score standardized using training-fold statistics only, which were recomputed independently in each fold and applied unchanged to the held-out fold. No post-hoc feature selection was performed, so no held-out information could enter the training pipeline.

Logistic regression, random forest, and support-vector classifiers used scikit-learn 1.7.2 with class-balanced weighting. The MLP baseline used PyTorch 2.10.0.dev20251114+cu128 and two hidden layers (7→64→32→2) with the same class-imbalance handling as the primary models. Supplementary Table S4 lists all baseline hyperparameters.

## 3. Results

### 3.1. Evaluation Metrics

Performance was evaluated using accuracy, balanced accuracy (BAcc), male recall, female recall, macro-F1 score, and female-positive AUC. Because the analytic cohort exhibits a 2.46:1 female-to-male imbalance (251 females versus 102 males), conventional overall accuracy would yield an optimistically biased estimate for any classifier that defaults to the majority class [42]. Balanced accuracy (BAcc) is therefore reported as the primary metric and is defined as the unweighted arithmetic mean of per-class recall

(5) $$\mathrm{BAcc}=\frac{1}{2}\left(\frac{\mathrm{TP}}{\mathrm{TP}+\mathrm{FN}}+\frac{\mathrm{TN}}{\mathrm{TN}+\mathrm{FP}}\right)=\frac{1}{2}\left({\mathrm{Recall}}_{M}+{\mathrm{Recall}}_{F}\right),$$

where female is taken as the positive class. TP, TN, FP, and FN denote true positives, true negatives, false positives, and false negatives, respectively. BAcc provides an unbiased performance summary under class imbalance and allows comparison across studies with differing sex ratios [43].

All primary results are presented as the mean ± standard deviation across the five folds. Pooled out-of-fold statistics are reported separately for descriptive purposes. Average precision (AP) was additionally computed with each sex as the positive class, and the Matthews correlation coefficient (MCC) was computed from the confusion matrix.

### 3.2. Baseline Comparison

To contextualize the tiered framework, the full-skull (Tier 1) and mandibular (Tier 2) pathways were evaluated against morphology-only baselines using the same five-fold protocol and identical fold assignments. The Tier 2 adult subgroup (n=259, ages 19–50) is included in the same table. However, its results were derived from the Tier 2 out-of-fold predictions rather than from a separately trained model.

**Figure 2:**
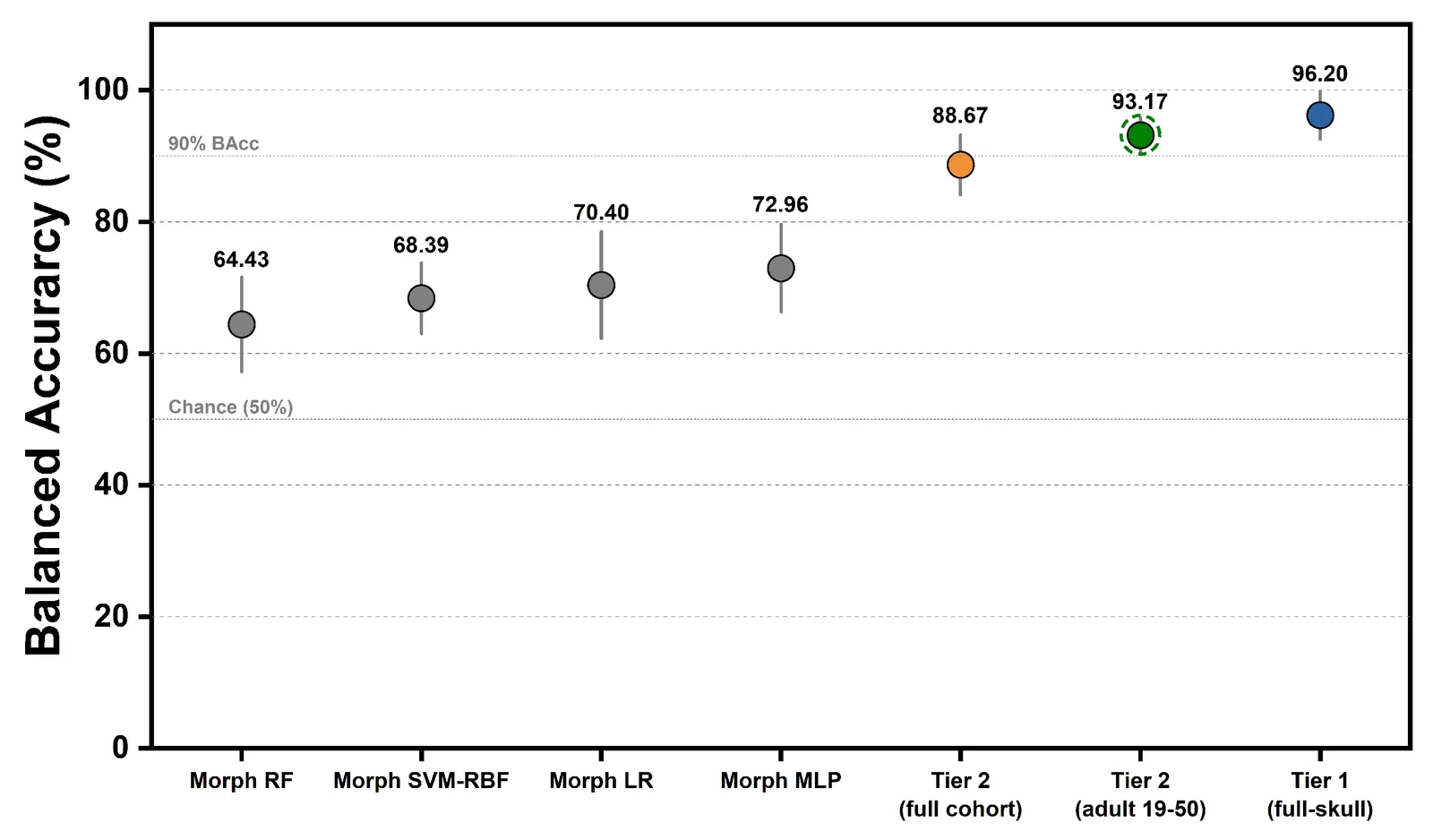
Performance comparison across the tiered framework (five-fold stratified cross-validation; mean balanced accuracy ±1 standard deviation). Gray, orange, green, and blue markers denote the morphometric baselines, Tier 2 full cohort, Tier 2 adults, and Tier 1, respectively. The dashed ring identifies the adult subgroup (ages 19–50, n=259), evaluated using the same folds as the full-cohort Tier 2 path. Morphometric baselines are the four multi-feature models; the volume-only baseline is in Table 3. RF, random forest; LR, logistic regression.

The primary results are summarized in Table 3 and Figure 2. Tier 1, which used $128^{3}$ grayscale full-skull CBCT volumes, achieved a mean balanced accuracy of 96.20%±3.66% and an accuracy of 96.60%±2.97%. The pooled recalls for male and female cases were 95.1% and 97.2%, respectively. Tier 2, based on $80^{3}$ mandibular binary masks, achieved a mean balanced accuracy of 88.67%±4.58% and an accuracy of 88.41%±5.00%. Its pooled recalls for male and female cases were 89.2% and 88.0%, respectively. The Tier 2 adult subgroup reached 93.17%±2.63% (accuracy 91.12%±2.71%), with every fold above 90% balanced accuracy and a marked recall asymmetry (pooled male 97.3%, female 88.7%). For the same 19–50 subgroup, the Tier 1 full-skull model reached 97.12%±2.89% BAcc (fold mean, with per-stratum pooled values in Supplementary Table S2), narrowing the full-skull advantage to a descriptive 3.95 percentage points over the mandible-only model. For this descriptive adult-subgroup analysis, the ± values denote the standard deviation across the five folds. No separate age-stratum hypothesis test was performed.

For context, the morphology-only baselines trained on mask-derived shape descriptors reached mean balanced accuracy in the 64 to 73% range (logistic regression 70.40%±8.14%, multilayer perceptron 72.96%±6.64%, random forest 64.43%±7.19%, support vector machine (SVM) 68.39%±5.36%). To test whether the mandibular pathway merely encodes overall size, a volume-only logistic regression (using total mandibular volume in ${\mathrm{mm}}^{3}$ as the single feature, with an identical five-fold split, train-fold standardization, and class balancing) reached 70.30%±8.22% balanced accuracy, 18.4 percentage points below the Tier 2 network (88.67%). In the 19–50 subgroup, the same volume-only baseline reached 71.37% balanced accuracy, remaining 21.8 percentage points below the Tier 2 adult subgroup result. All four multi-feature baselines and this single-feature model fell substantially below Tier 2. Although mandibular volume is sex-dimorphic, it explains only part of the discriminative signal. The volume-only baseline (70.30%) was nearly identical to the seven-feature morphometric logistic regression (70.40%), indicating that these predefined scalar descriptors added little beyond total volume. That the Tier 2 mask model substantially exceeded both scalar baselines shows it was not merely a volume classifier, but used spatial information in the mandibular-region mask not captured by these descriptors. This spatial information may include boundary geometry, alveolar and tooth-socket geometry, and patterns related to segmentation or acquisition, and should not be attributed to isolated mandibular bone shape alone.

Within Tier 2, the no-SE backbone reached 88.67%±4.58% against 85.84%±6.88% for the SE-enabled variant. The difference was not statistically distinguishable (paired McNemar p=0.672, bootstrap 95% CI [−2.09%,+5.77%] spanning zero). The no-SE variant also showed lower fold-level variance, and was therefore retained as the Tier 2 architecture for all subsequent analyses. The SE variant is reported in full in Supplementary Note S2.

The mean female-positive AUC for Tier 1, Tier 2, and the Tier 2 adult subgroup was 0.961, 0.910, and 0.938, with pooled out-of-fold receiver operating characteristic (ROC) curves provided in Supplementary Figure S2. By construction, the pooled-curve AUCs in Figure S2 differ slightly from these fold-mean values. Tier 1 exceeded Tier 2 on this cohort, and the mandible-only path restricted to the adult subgroup fell between the two at 0.938.

The adult-subgroup gain over the full-cohort Tier 2 was asymmetric across sex. Pooled male recall rose from 89.2% in the full cohort to 97.3% in the adult subgroup, whereas pooled female recall stayed near constant (88.0% to 88.7%). Age-stratified inspection (Table 4) localized this asymmetry to the young adult and middle adult strata, where male recall reached 98.0% and 95.7%. This asymmetry is reported as observed, and sex-specific recall should accompany balanced accuracy in operational forensic settings.

### 3.3. Age-Stratified Performance

**Figure 3:**
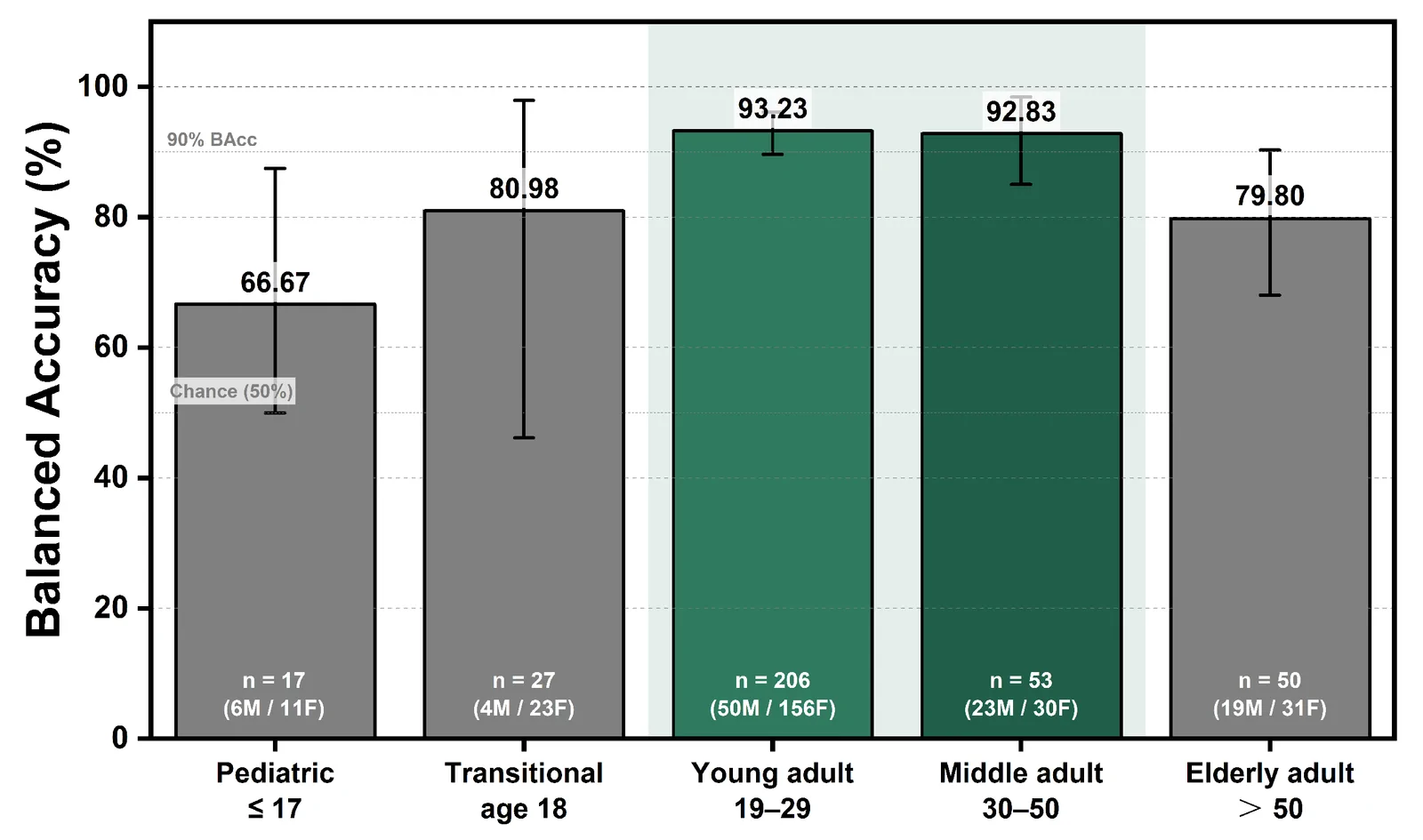
Tier 2 balanced accuracy across five pre-defined age strata, reported as pooled out-of-fold values. Error bars represent 95% bootstrap confidence intervals based on 5000 case-level resamples. Bar labels indicate the sample size (*n*) and male-to-female distribution within each stratum. The shaded 19–50 age range denotes the Tier 2 adult subgroup.

To characterize performance across age groups, Table 4 and Figure 3 report the pooled out-of-fold sample composition and Tier 2 performance for each of the five pre-defined age strata.

Between the pediatric (≤17) and young-adult (19–29) strata, the small transitional stratum comprising 18-year-old individuals (n=27, 4 male and 23 female) exhibited intermediate Tier 2 performance. Its balanced accuracy was 80.98%, overall accuracy was 85.19%, and male and female recalls were 75.0% and 87.0%, respectively. This stratum represents the transition to adulthood, during which craniofacial maturation may still vary appreciably among individuals. It was therefore reported separately to avoid combining developmentally heterogeneous cases.

The young-adult stratum (19–29) contained the largest number of cases (n=206) and represented mature mandibular dimorphism, achieving the highest pooled balanced accuracy of 93.23%. The middle-adult stratum (30–50) produced a closely comparable value of 92.83% for n=53. Together, these strata constitute the 19–50 prime forensic demographic reported as the Tier 2 adult subgroup, with an aggregate balanced accuracy of 93.17%±2.63% (Section 3.2). In contrast, the pediatric stratum (≤17) had the lowest pooled balanced accuracy, at 66.67% for n=17, whereas the elderly stratum (>50) exhibited intermediate-to-low performance, at 79.80% for n=50.

Error rates across strata are described in Section 3.8 and discussed in Section 4. In a post-hoc model of paired misclassification, the odds of misclassification remained higher with the mandible-only input after adjustment for age group and sex (odds ratio 3.78, 95% CI 2.02–7.09). The tier × age-group interaction was not statistically significant (p=0.47), although the small number of Tier 1 errors limits this test (Supplementary Table S10). An age-only classifier reached a BAcc of 58.13%, and age-standardized BAcc was 96.28% for Tier 1 and 89.05% for Tier 2. These analyses supplement the matched comparison but do not show that age-related confounding in the CNNs has been eliminated.

### 3.4. Cross-Fold Variability

Table 5 presents the fold-wise performance of Tier 1, Tier 2, and the Tier 2 adult subgroup, with the corresponding per-fold values displayed in Supplementary Figure S3. Across the five folds, Tier 1 balanced accuracy ranged from 89.86% to 99.02%. Four folds exceeded 96%, whereas Fold 3 produced a lower value of 89.86%, indicating fold-level variability at the current sample size. Tier 2 balanced accuracy ranged from 82.86% to 95.50%. Fold 3 again yielded the lowest value, at 82.86%, while the other four folds exceeded 86%. A fold-composition audit (Supplementary Table S7) showed that Fold 3 was comparable to the other folds in sex ratio, median age, and age-stratum composition, indicating that its lower performance was not explained by demographic imbalance. To further assess dependence on the fold split, the complete five-fold training and evaluation was repeated under two additional cross-validation (CV)/model seed settings (Supplementary Table S8). Tier 2 reached fold-mean balanced accuracies of 88.27% ± 2.07% and 86.04% ± 3.94% under these settings, compared with 88.67% ± 4.58% under the primary seed. Across the three settings, fold-mean balanced accuracy ranged from 86.04% to 88.67%, and the lower-performing fold shifted across seeds, indicating that the primary result was not specific to a single seed setting while some residual split and training-seed variability remained. For the Tier 2 adult subgroup, balanced accuracy ranged from 90.26% to 97.14%, with values above 90% in every fold.

### 3.5. Statistical Comparisons

Table 6 summarizes paired statistical comparisons between key model configurations, based on case-level OOF predictions. For each bootstrap replicate *b*, paired BAcc differences were computed as

(6) $$\Delta {\mathrm{BAcc}}^{\left(b\right)}={\mathrm{BAcc}}_{1}^{\left(b\right)}-{\mathrm{BAcc}}_{2}^{\left(b\right)},\;b=1,\ldots ,B,$$

where each replicate resamples cases with replacement under stratification by sex label and by case-level pairing across tiers, with B=5000. The 95% CI for ΔBAcc was computed as the [2.5,97.5] percentile interval of $\Delta {\mathrm{BAcc}}^{\left(b\right)}$.

For the tier-wise comparison matched by case under shared fold assignments, paired McNemar testing [44] on case-level predictions yielded $p=1.54\times 10^{-5}$, with 37 cases correct by Tier 1 only and 8 correct by Tier 2 only. The stratified paired bootstrap [45] 95% interval for the balanced-accuracy difference excluded zero, confirming a distinguishable advantage of full-skull over mandible-only input on this cohort.

The differences in average precision between the inputs were small and imprecisely estimated. Average precision was 0.852 versus 0.843 with males as the positive class and 0.962 versus 0.936 with females as the positive class, with 95% CIs including zero for both paired differences. The Matthews correlation coefficient favored Tier 1 (0.918 versus 0.738, difference 0.180, 95% CI 0.104–0.261, Supplementary Table S9 and Figure S4). The comparison therefore supports a full-skull advantage in BAcc and MCC, whereas the AP difference remains uncertain. In the Tier 1 male-positive curve, three females with near-maximal male scores (P(male)>0.99) were among the four highest-scored cases, which lowers precision at the start of the curve (Figure S4a).

The SE-ablation comparison within Tier 2 (p=0.672, bootstrap CI [−2.09%,+5.77%], 27 and 23 discordant cases) did not exclude zero, indicating no single-branch advantage from SE on this cohort. These paired tests quantify prediction-level differences and complement the accuracy summaries in Section 3.2.

### 3.6. Disentanglement of Anatomical Scope, Modality, and Resolution

The Tier 1 versus Tier 2 comparison in Section 3.2 confounds differences in anatomical scope (full skull versus mandible only), input modality (grayscale intensities versus binary mask), and spatial resolution ($128^{3}$ versus $80^{3}$). To isolate each factor, we trained two intermediate configurations under the identical five-fold protocol (seed 42). These comprised a full-skull grayscale volume downsampled to $80^{3}$ (Tier 1-80), and a mandible-only grayscale volume masked to the segmented bone (Tier 2-Gray). With Tier 1 and Tier 2, these form a four-configuration matched ablation in which scope, modality, and resolution each vary one at a time (Table 7).

Because adjacent configurations differ by a single factor, the Tier 1 → Tier 2 gap decomposes into three additive paired contrasts (Table 8). Anatomical scope contributed most. At matched grayscale modality and $80^{3}$ resolution, restricting the input from the full skull to the mandible reduced balanced accuracy by 16.02 percentage points (95% bootstrap CI [−20.67,−11.61], McNemar exact $p=5.87\times 10^{-15}$). Resolution had no detectable effect. Downsampling the full skull from $128^{3}$ to $80^{3}$ changed balanced accuracy by −0.98 percentage points (95% bootstrap CI [−3.25,+1.09], p=0.77). Modality reversed the expected direction. Within the mandibular region, the binary-mask input outperformed the grayscale-intensity input by 9.48 percentage points (95% bootstrap CI [+5.55,+13.71], $p=1.31\times 10^{-5}$). By construction, the three contrasts sum to the overall Tier 1 → Tier 2 difference of −7.52 percentage points (88.63−96.15). The exact McNemar tests were not adjusted for multiple comparisons. However, given the magnitude of the primary *p*-values, applying a Holm–Bonferroni correction would not change which contrasts reached statistical significance.

Within this single-center cohort evaluated by internal cross-validation, anatomical scope, rather than spatial resolution, primarily accounted for the performance difference between the full-skull and mandible-only inputs. When the anatomical scope was restricted to the mandible, the binary-mask input outperformed the grayscale-intensity input. Potential mechanisms underlying this reversal in modality performance are discussed in Section 4.

With the lower-teeth label added, Tier 2 reached a fold-mean BAcc of 86.92% ± 5.21%, compared with 88.67% ± 4.58% for the original input. The paired difference in pooled BAcc was −1.84 percentage points (95% CI −5.77 to 1.79). Supplementary Table S11 gives the full results, including sex-specific recall. This single matched comparison did not provide clear evidence of a difference in BAcc between the inputs. Both inputs retain the alveolar process and tooth sockets, so the comparison does not separate alveolar from basal-bone contributions.

### 3.7. Grad-CAM Visualization

**Figure 4:**
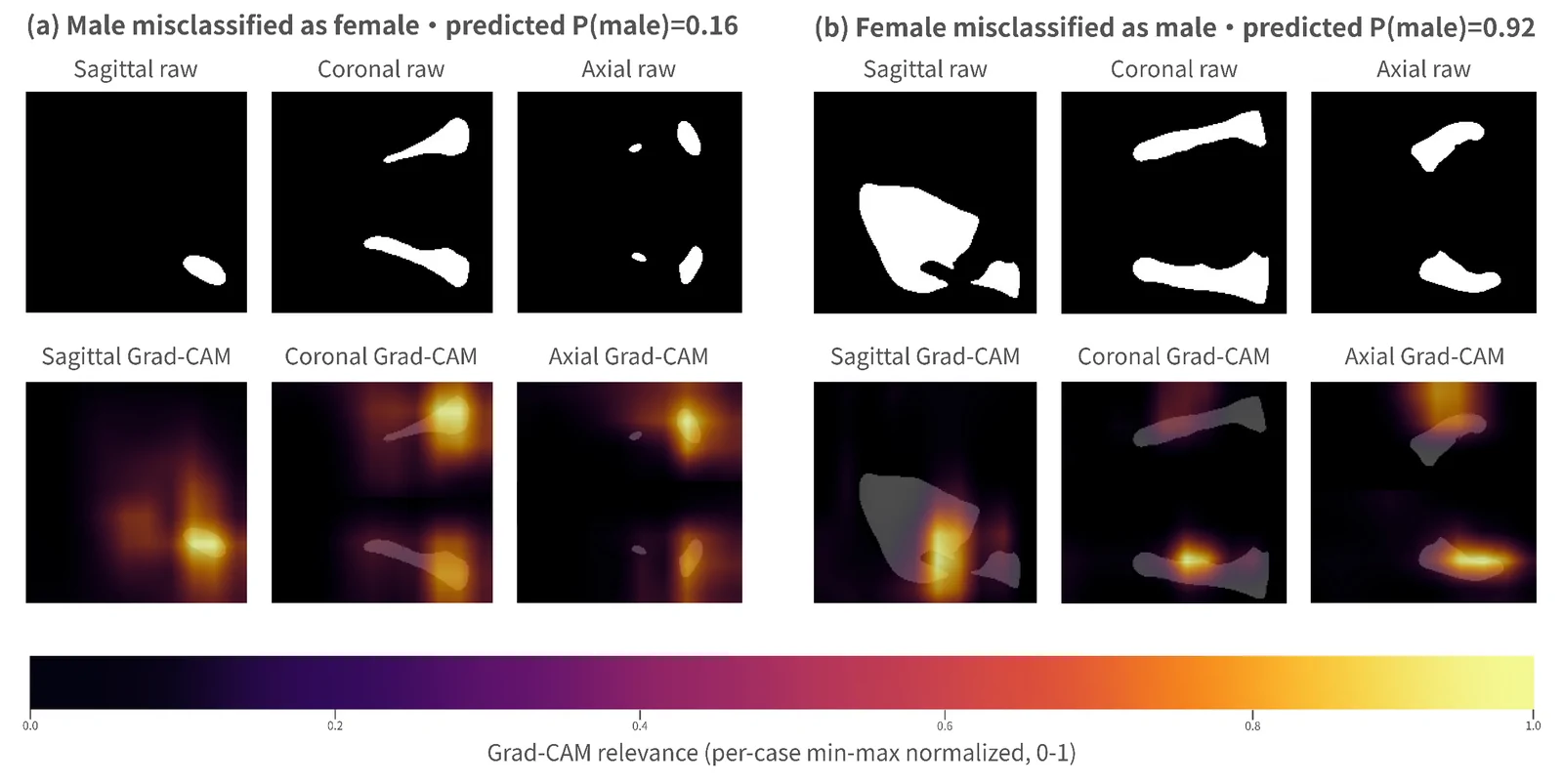
Representative Tier 2 gradient-weighted class activation mapping (Grad-CAM) misclassifications from the no-SE mandibular model. Panel (**a**) shows a 9-year-old male misclassified as female (predicted P(male) = 0.16). Panel (**b**) shows a 24-year-old female misclassified as male (predicted P(male) = 0.92). Each panel gives three orthogonal views, with the raw mandibular mask on top and the Grad-CAM overlay below. Maps are min-max normalized within each case, so absolute relevance is not comparable across panels. A contact sheet of eight correct classifications appears in Supplementary Figure S1.

To determine whether the model relied on anatomically plausible features, three-dimensional gradient-weighted class activation mapping (Grad-CAM) [46] was generated for 10 representative cases from the no-SE mandibular (Tier 2) branch. Figure 4 presents two illustrative misclassified cases, one male and one female, while the remaining eight correctly classified cases are provided in Supplementary Figure S1. The final convolutional block was selected as the target layer. The rerun reproduced the saved out-of-fold logits to numerical precision, with a mean $\left|\Delta p_{\mathrm{male}}\right|$ of $2.53\times 10^{-8}$.

For high-confidence correct classifications, activation was concentrated primarily around the gonial angle, posterior mandibular body, and ramus, all of which are regions with pronounced sexual dimorphism. By comparison, activation was more diffuse in near-threshold cases, suggesting a weaker discriminative signal. In both misclassified cases, the activation remained focal. The maps show where the evidence was concentrated but not why the prediction was incorrect. Some cases also exhibited partial activation over the alveolar region (Section 2.1). The maps are coarse and cannot separate alveolar from basal-bone contributions.

These activation patterns are qualitatively consistent with the discriminative regions identified by Shimkus et al. [25] using Croatian mandibular point clouds. Although the Grad-CAM results support this consistency, they cannot independently exclude artifact-driven learning. Moreover, the resulting attributions are coarse and should not be interpreted as precise anatomical localization.

### 3.8. Error Analysis

Error analysis of the Tier 2 out-of-fold predictions identified 41 errors among 353 cases (11.61%). Error rates were highest in the ≤17 (4 of 17) and >50 (10 of 50) strata, whereas the 19–50 adult demographic had an error rate of 8.88% (23 of 259) and, as the largest group, contained most errors. For Tier 1, the error count was far lower, at 12 among 353 cases (3.40%), comprising 3 boys aged 6–9 years, 8 cases in the 19–29 stratum, and 1 case older than 50 years.

Comparing the two tiers on the same paired cases, the set misclassified by Tier 2 substantially exceeded that misclassified by Tier 1 (41 against 12). Of all 353 paired cases, 304 (86.1%) were correct under both tiers and 4 (1.1%) under neither. The 4 cases misclassified by both tiers were three boys aged 6–9 years and one 25-year-old man, all classified as female.

**Figure 5:**
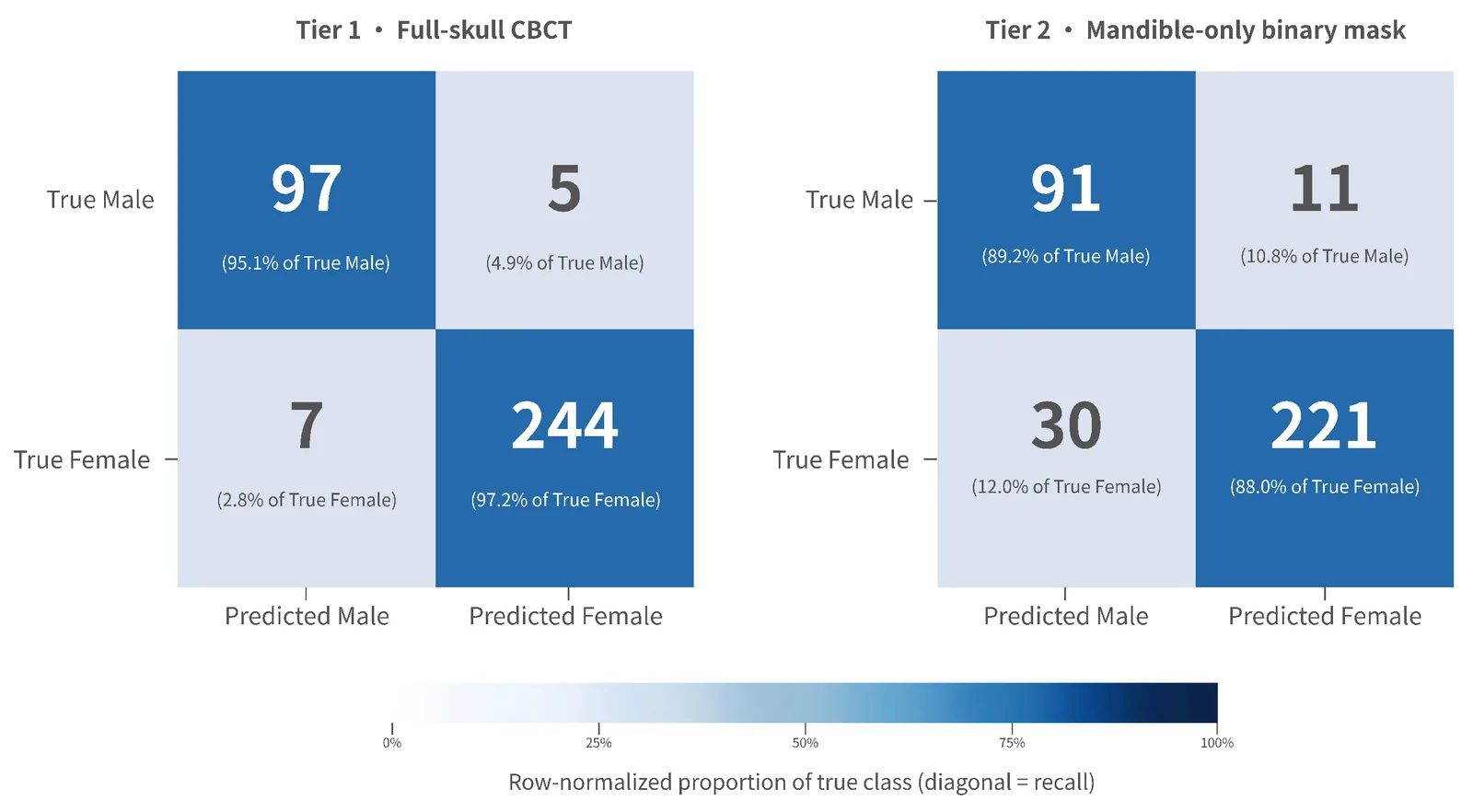
Pooled out-of-fold confusion matrices for Tier 1 (full-skull CBCT, **left**) and Tier 2 (mandible-only binary mask, **right**). Female is the positive class, N = 353. Each cell shows the count and the within-row percentage, so diagonal values equal recall. Both matrices share one color scale, and the panel titles identify the tier. These are pooled values, and Table 3 reports the fold-mean estimates.

The Tier 2 pooled out-of-fold confusion matrix across all 353 cases, with female as the positive class, was TN=91, FP=11, FN=30, TP=221, giving pooled male recall 89.2% and female recall 88.0%. The Tier 1 matrix was TN=97, FP=5, FN=7, TP=244, giving pooled male recall 95.1% and female recall 97.2%. Both are visualized in Figure 5.

### 3.9. Computational Cost

To quantify the computational footprint, inference latency and GPU memory usage were benchmarked using PyTorch at 32-bit floating-point (FP32) precision on a single NVIDIA RTX 5070 Ti GPU (NVIDIA, Santa Clara, CA, USA). Latency was averaged over 100 forward passes following 10 warm-up iterations, with a batch size of 1. For Tier 2, using an 80×80×80 mandibular input and excluding preprocessing and input/output operations, the no-SE backbone required 3.36ms per case and reached a peak GPU memory usage of 138.81MB. For Tier 1, using a 128×128×128 full-skull input, the same backbone required approximately 11.4ms per case, with a peak memory usage of 384MB.

## 4. Discussion

This study investigated a tiered framework that applied a shared backbone and training protocol to full-skull and mandible-only CBCT inputs from 353 Chinese Han cases. Under matched experimental conditions, the full-skull Tier 1 pathway exceeded the performance of the mandible-only Tier 2 pathway by approximately 7.5 percentage points. Instead of treating this difference as the combined result of anatomical scope, input modality, and resolution, the four-configuration ablation presented in Section 3.6 distinguished the contribution of each factor. This section discusses the resulting decomposition in conjunction with the age-stratified findings.

The decomposition demonstrated that the full-skull advantage was primarily determined by anatomical scope rather than resolution. With input modality and resolution held constant, limiting the anatomical coverage to the mandible reduced balanced accuracy by approximately 16 percentage points. In comparison, reducing the linear grid size from 128 to 80 produced no detectable effect. This is consistent with the full skull carrying a dimorphic signal beyond the mandible (the supraorbital region, mastoid processes, and overall cranial size) that a mandible-only crop necessarily discards. The negligible resolution effect indicates that the discriminative morphology survives at $80^{3}$. This resolution contrast was evaluated on the full-skull input. Mandibular resolution was not separately varied, so the robustness of the mandible-only path to downsampling is inferred rather than directly tested.

More unexpectedly, the modality axis acted in the opposite direction. Within the mandible, the binary mask exceeded the grayscale crop by about 9.5 percentage points. This binary-mask advantage should not be read as evidence that grayscale CBCT information is intrinsically uninformative. At full-skull scope, grayscale volumes preserve broad craniofacial morphology and overall bone and background structure, which provides a strong shape signal. Once the scope is restricted to the mandible, uncalibrated intra-bone intensity variation, the alveolar component, and local acquisition artifacts may dilute the boundary and shape signal, or make the masked grayscale representation harder to optimize at the present cohort size. The observed reversal may therefore reflect suppression of scanner-dependent intensity variation, a stronger emphasis on mandibular geometry, or easier optimization of a shape-dominant representation. The Grad-CAM activation over the gonial angle and ramus (Section 3.7) is compatible with, but does not demonstrate, these explanations. It should be read as an effect observed under this single-center cohort and preprocessing scheme, not as a general superiority of binary over grayscale input.

A relevant point of comparison is the study by Shimkus et al. [25], which reported 93% cross-validation accuracy and 92% independent-test accuracy using PointNet++ on three-dimensional mandibular point clouds from a Croatian population (Supplementary Table S1). However, several differences in study design limit direct comparison. Their cohort was balanced by sex (127/127), whereas the present cohort was imbalanced (102/251). Moreover, the age distribution of their cohort was not reported, while our participants ranged from 6 to 78 years and included pediatric and geriatric cases, in which mandibular sexual dimorphism is less pronounced. The studies also differed in their input representations and evaluation protocols, placing them in distinct methodological settings.

Within the 19–50 demographic, the Tier 2 adult subgroup achieved a balanced accuracy of 93.17%. Further stratification into the 19–29 and 30–50 age ranges yielded closely comparable performance. In contrast, mandible-only performance was substantially lower in the pediatric (≤17) and elderly (>50) strata. Within this adult subgroup, recall was markedly higher for males than for females (97.3% versus 88.7%), which also lifts balanced accuracy (93.17%) above raw accuracy (91.12%). Because the same reweighting left recall nearly symmetric in the full cohort (89.2% versus 88.0%), this asymmetry more likely reflects the age restriction retaining skeletally mature males with more strongly dimorphic mandibles rather than the reweighting itself, which at most compounds it.

The pattern of errors suggests how age may influence the mandible-only model. Sex differences characteristic of the adult mandible develop mainly during and after puberty [41,47]. Consistent with this, every Tier 2 error in the ≤17 stratum was a boy classified as female (male recall 2/6, female recall 11/11), and the full-skull input showed the same pattern (3/6 and 11/11). In older adults, age-related remodeling alters mandibular shape [21], and tooth loss is followed by resorption of the alveolar bone. In the >50 stratum, Tier 2 errors occurred in both directions (male recall 15/19, female recall 25/31). Excluding the tooth labels does not establish robustness to crown wear or to postmortem tooth loss or displacement, which may affect segmentation. Antemortem tooth loss can also alter alveolar morphology. Performance under these conditions was not evaluated in this study. In a sex-adjusted model, Tier 2 errors were more likely above 50 years than at 19–50 years (odds ratio 2.61, 95% CI 1.15–5.91), whereas the estimate for ≤18 years was imprecise (2.26, 0.94–5.45, Supplementary Table S10). These strata are small, and dental status was not assessed, so these explanations remain plausible rather than demonstrated.

The same age dependence appeared on the full-skull pathway but was milder. The largest tier-wise gap fell in the transitional age-18 stratum, where Tier 1 classified all 27 cases correctly while Tier 2 reached 80.98%, and in the elderly stratum, where the mandibular pathway dropped to 79.80% against near-ceiling full-skull accuracy (Supplementary Table S2). Because these strata were small, the differences are exploratory. They may reflect craniofacial regions outside the mandible that remain informative.

Taken together, these analyses support a tiered inference design with the full-skull pathway when the craniofacial region is intact, and the mandible-only pathway when only mandibular evidence is available, with performance dependent on demographic composition. However, no automatic triage or routing was implemented or evaluated. The tiers denote which input is available rather than a learned selector.

In casework, the age and sex of unidentified remains are usually unknown at the outset. Neither network received age as an input, so the full-cohort estimates describe internal performance in this cohort without explicit age input. They do not validate casework involving remains of unknown age. The 19–50-year estimate should not be extrapolated to other age groups, and applying it would require an independent age assessment. Each input was trained with a single all-age objective (6–78 years) to keep the comparison matched. This matching supports the within-cohort comparison but does not eliminate age effects within either model. An age-stratified decision rule explored during development gave no robust gain under nested rule selection (Supplementary Note S1), and age-specific training was not evaluated. An operational workflow that selects models using estimated age would additionally require validation of age-assessment errors.

The network outputs are uncalibrated softmax scores (Section 2.4). They have not been established as calibrated case-level probabilities and should not be interpreted as likelihood ratios. Balanced accuracy is a cohort-level summary rather than the probability that an individual classification is correct. The present results describe the discrimination performance of each input in this reference sample. Any likelihood-ratio interpretation would require a separately specified and independently validated evidential framework, which was not evaluated here. The model output should not be presented as standalone expert evidence.

The study has the following limitations.

(1) The cohort is single-center, Chinese Han, and sex-imbalanced (102 male, 251 female). Cross-population and cross-scanner transferability of skeletal sex-estimation models is known to degrade under population and acquisition shift [48], and no independent external test set was available to assess this here. An expansion to approximately 600 cases with balanced sex composition and multi-center sourcing is underway as the essential next step before operational deployment.
(2) The pediatric (≤17, n=17) and elderly (>50, n=50) strata are small. Their results are exploratory and should be read as indicative rather than definitive.
(3) The primary tier-wise comparison used a single seed-42 stratified five-fold split, applied identically to both tiers to preserve the paired comparison. Both tiers reached their lowest balanced accuracy in Fold 3 (Tier 1 89.86%, Tier 2 82.86%). A fold-composition audit found Fold 3 demographically comparable to the other folds in sex ratio, median age, and age-stratum composition. Because both tiers share the same fold assignment, this concurrent drop most likely reflects case-level variability within that split rather than two separate failures. A repeated-CV sensitivity analysis under two additional CV/model seed settings was performed for Tier 2 only (Section 3.4 and Supplementary Table S8), yielding a 2.63 percentage-point range in fold-mean balanced accuracy across seeds. This indicates that the mandible-only estimate was not unique to the seed-42 split, while some residual split and training-seed variability remained. Because Tier 1 was not repeated under additional seeds, the paired full-skull-versus-mandible gap should be read as an internal estimate under the primary matched split. Larger repeated-CV or external validation cohorts would further refine this uncertainty estimate.
(4) The Tier 2 input of the main analysis is the automatically segmented mandibular representation. Only the mandible label was retained, and the separately labeled teeth were not merged. The alveolar process and socket geometry, nevertheless, remain and may contribute to the prediction. Adding the lower-teeth label did not provide clear evidence of a difference in BAcc (Section 3.6). The study design does not separate the contributions of the ramus and body, the alveolar process, and the socket geometry. The coarse Grad-CAM maps cannot distinguish these contributions either. The mandible-only results should therefore be interpreted as the performance of this representation as a whole. Robustness to crown wear, tooth loss or displacement, and antemortem alveolar resorption was not evaluated.

## 5. Conclusions

Skeletal sex estimation reaches high accuracy within single cohorts. Yet reported results rarely transfer across studies, because cohorts, input representations, and evaluation protocols differ. The practical cost of relying on the mandible alone has therefore remained unclear. This study quantified, under matched conditions on a single Chinese Han cohort of 353 cases, how much sex-estimation performance decreases when the CBCT input is reduced from the full skull to the mandible alone. Both inputs were processed through an identical backbone, fold assignment, and training protocol, with model capacity held constant across the two paths, so that the design isolates the effect of anatomical input rather than of model differences. A case-level paired bootstrap placed the full-skull advantage at 4.55–10.50 percentage points. A four-configuration ablation attributed this gap primarily to anatomical scope rather than resolution, with the binary mask unexpectedly outperforming grayscale intensities once scope was fixed to the mandible, on this uncalibrated-CBCT cohort.

Within this cohort and under the matched evaluation protocol, the full-skull input performed better overall. The mandible-only model achieved a fold-mean BAcc of 93.17% in adults aged 19–50 years (n=259). This subgroup estimate should not be extrapolated to the other age groups. Its pooled BAcc was 80.98% at age 18, 66.67% at ≤17 years, and 79.80% above 50 years. The full-skull model retained a pooled BAcc of at least 96% in each stratum from age 18 upward and reached 75.00% at ≤17 years. Estimates in the smaller age strata are exploratory. These findings support the within-cohort comparison. External validation is required before operational forensic use, and the model output should not serve as standalone expert evidence.

## Figures and Tables

**Table 1:** Cohort summary. IQR, interquartile range; M/F, male/female; SE, squeeze-and-excitation.

Item	Value
Initially screened cases (mandibular masks produced)	359
Excluded at quality control	6
Age, median (IQR)	22 (20–35), full range 6–78 years
Total cases	353
Male	102
Female	251
Pediatric ≤ 17 (M/F)	6/11 (n=17)
Transitional age 18 (M/F)	4/23 (n=27)
Adult 19–50 (M/F)	73/186 (n=259)
Elderly > 50 (M/F)	19/31 (n=50)
Cross-validation	5-fold stratified
Input size	Tier 1 128×128×128 (grayscale CBCT), Tier 2 80×80×80 (binary mask)
Backbone parameters	572,338 (no-SE, retained for all tiers)/583,218 (SE variant, ablation only)

**Table 2:** Architecture specification of the lightweight 3D CNN backbone.

Layer	Type	Output Size	Channels	Kernel	Stride
Input	—	80×80×80	1	—	—
Block 1	2×(Conv3D+BN+ReLU) + MaxPool	40×40×40	16	3×3×3	1
Block 2	2×(Conv3D+BN+ReLU) + MaxPool	20×20×20	32	3×3×3	1
Block 3	2×(Conv3D+BN+ReLU) + MaxPool	10×10×10	64	3×3×3	1
Block 4	Conv3D+BN+ReLU + AdaptiveAvgPool	2×2×2	128	3×3×3	1
Flatten	—	1024	—	—	—
FC1	Linear + ReLU + Dropout(0.5)	128	—	—	—
FC2	Linear + ReLU + Dropout(0.3)	32	—	—	—
Output	Linear	2	—	—	—

Note: MaxPool uses kernel 2, stride 2. Output sizes shown are for the 80^3^ Tier 2 input. Tier 1 (128^3^) follows the same topology with proportionally larger intermediate dimensions. The no-SE variant (572,338 parameters) was retained as the final architecture for both tiers.

**Table 3:** Baseline comparison and ablation results (five-fold mean ± standard deviation (SD)).

Setting	Acc (%)	BAcc (%)	Male Recall	Female Recall	Macro-F1	AUC
Tier 1 (full-skull)						
(N=353, 572,338 params)	96.60±2.97	96.20±3.66	0.952±0.059	0.972±0.023	0.957±0.033	0.961±0.028
Tier 2 (mandible)						
(no-SE, N=353)	88.41±5.00	88.67±4.58	0.893±0.053	0.880±0.057	0.870±0.048	0.910±0.045
Tier 2 subgroup						
(ages 19–50, n=259)	91.12±2.71	93.17±2.63	0.975±0.034	0.889±0.034	0.910±0.030	0.938±0.022
Morphometric random forest	74.65±5.17	64.43±7.19	0.403±0.128	0.885±0.030	0.653±0.077	0.689±0.096
Morphometric SVM (RBF)	78.59±4.27	68.39±5.36	0.443±0.101	0.925±0.042	0.700±0.061	0.770±0.055
Morphometric logistic regression	70.42±5.27	70.40±8.14	0.704±0.161	0.706±0.040	0.672±0.069	0.745±0.122
Morphometric MLP	73.22±4.55	72.96±6.64	0.724±0.146	0.734±0.058	0.702±0.056	0.772±0.078
Volume-only logistic regression	71.67±5.35	70.30±8.22	0.669±0.166	0.737±0.044	0.679±0.071	0.750±0.089

Note: Higher is better. Acc, accuracy; BAcc, balanced accuracy; AUC, female-positive area under the receiver operating characteristic curve; SVM, support vector machine; RBF, radial basis function; MLP, multilayer perceptron; Macro-F1, macro-averaged F1 score. Training follows Section 2.4. Volume-only logistic regression uses total mandibular volume (mm^3^) as its sole feature, with the same five-fold split and standardization as the other baselines.

**Table 4:** Age-stratified composition and pooled out-of-fold (OOF) performance (Tier 2).

Age Stratum	*n* (M/F)	Accuracy	BAcc ↑	Male Recall	Female Recall	Errors (Rate)
Pediatric ≤ 17	17 (6/11)	76.47%	66.67%	33.3%	100.0%	4 (23.53%)
Transitional age 18	27 (4/23)	85.19%	80.98%	75.0%	87.0%	4 (14.81%)
Young adult 19–29 years	206 (50/156)	90.78%	93.23%	98.0%	88.5%	19 (9.22%)
Middle adult 30–50 years	53 (23/30)	92.45%	92.83%	95.7%	90.0%	4 (7.55%)
Elderly > 50	50 (19/31)	80.00%	79.80%	78.9%	80.6%	10 (20.00%)
Pooled OOF total	353 (102/251)	88.39%	88.63%	89.2%	88.0%	41 (11.61%)

Note: Per-stratum values summarize Tier 2 pooled out-of-fold predictions. ↑ indicates higher BAcc is better. The pooled-total row covers all 353 cases (fold-mean ± SD, 88.67% ± 4.58%; Table 3). The 19–50 window combines the young and middle adult strata for the Tier 2 adult subgroup (*n* = 259), reported as fold-mean statistics in Section 3.2.

**Table 5:** Fold-wise balanced accuracy across the tiered framework.

Fold	Tier 1 BAcc (%) ↑	Tier 2 BAcc (%) ↑	Tier 2 Adult Subgroup BAcc (%) ↑
1	99.02	89.10	94.12
2	97.62	86.90	90.26
3	89.86	82.86	92.68
4	96.50	89.00	91.65
5	98.00	95.50	97.14

Note: The Tier 2 adult subgroup column is computed on the 19–50 adult subset within each fold (subset sizes of approximately 52 cases per fold). ↑ indicates that higher BAcc is better.

**Table 6:** Paired statistical comparisons based on case-level OOF predictions.

Comparison	McNemar *p*	Bootstrap 95% CI (BAcc Diff)	Interpretation
no-SE vs. SE	0.672	[−2.09%,+5.77%]	Not distinguishable
(single fixed window)			
Tier 1 vs. Tier 2	$1.54\times 10^{-5}$	[+4.55%,+10.50%]	Tier 1 exceeds Tier 2
(full skull vs. mandible)			

Note: Discordant counts (correct by A only/correct by B only) for Tier 1 vs. Tier 2 were 37/8, and those for no-SE vs. SE were 27/23. The McNemar exact binomial test evaluates asymmetric disagreement on paired cases, not the balanced-accuracy difference. Bootstrap intervals use 5000 stratified paired resamples (stratified by sex label, paired by case). CI, confidence interval.

**Table 7:** Per-tier metrics for the four-configuration matched ablation (five-fold stratified cross-validation, seed 42).

Configuration	Scope	Modality	Resolution	N	Pooled BAcc (%)	Fold-Mean BAcc ± SD (%)	AUC	Male Recall
Tier 1	Full skull	Grayscale	$128^{3}$	353	96.15	96.20±3.66	0.950	0.951
Tier 1-80	Full skull	Grayscale	$80^{3}$	353	95.17	95.25±4.67	0.948	0.931
Tier 2-Gray	Mandible only	Grayscale × mask	$80^{3}$	353	79.15	79.23±4.53	0.801	0.794
Tier 2	Mandible only	Binary mask	$80^{3}$	353	88.63	88.67±4.58	0.907	0.892

Note: AUC and male recall are pooled out-of-fold values. Balanced accuracy is reported both as the pooled value and as the fold-mean ± SD over the five folds.

**Table 8:** Pairwise disentanglement of resolution, anatomical scope, and modality effects on balanced accuracy.

Axis	Pair (A→B)	Δ BAcc (pp)	95% Bootstrap CI (pp)	McNemar Discordant (*A*:*B*)	McNemar *p*
Resolution	T1 → T1-80	−0.98	[−3.25,+1.09]	7:5	0.77
Anatomical scope	T1-80 → T2-Gray	−16.02	[−20.67,−11.61]	64:4	$5.87\times 10^{-15}$
Modality (mandible only)	T2-Gray → T2	+9.48	[+5.55,+13.71]	12:45	$1.31\times 10^{-5}$

Note: Δ is the paired difference in pooled balanced accuracy (*B* − *A*). Negative values indicate lower BAcc for *B*. pp, percentage points.

## Data Availability

The original contributions presented in the study are included in the article/Supplementary Materials. Further inquiries can be directed to the corresponding author.

## Supplementary Materials

The following supporting information can be downloaded at https://www.mdpi.com/article/10.3390/biology15201891/s1. Supplementary Figure S1. Grad-CAM contact sheet: eight representative Tier 2 correct classifications (Panels a–h). Supplementary Figure S2. ROC curves for Tier 1, Tier 2, and the Tier 2 adult subgroup across pooled out-of-fold predictions. Supplementary Note S1. Exploratory age-stratified dual-branch fusion analysis and nested rule-selection stress test (archival). Supplementary Note S2. Full SE ablation for the Tier 2 mandibular path. Supplementary Note S3. ResNet3D-18 contextual reference (architecture comparison with cohort and schedule caveats). Supplementary Figure S3. Per-fold balanced accuracy across the tiered framework (Tier 1/Tier 2/Tier 2 adult subgroup). Supplementary Table S1. Summary of representative mandibular sex estimation studies (cross-study literature comparison). Supplementary Table S2. Tier-wise age-stratified and adult-subgroup performance summaries. Supplementary Table S3. CBCT acquisition audit summary (scanner configuration and scan-parameter distribution). Supplementary Table S4. Hyperparameters of the four morphometric baseline classifiers and the volume-only baseline. Supplementary Table S5. Locked age-stratified fusion weights and thresholds (exploratory, archival), (presented within Supplementary Note S1). Supplementary Table S6. Tier 2 fixed-window single-branch performance, no-SE versus SE (presented within Supplementary Note S2). Supplementary Table S7. Fold composition audit for the seed-42 five-fold split (support for Section 3.4 cross-fold variability and Limitation 3). Supplementary Table S8. End-to-end repeated five-fold sensitivity for Tier 2. Supplementary Figure S4. Pooled out-of-fold precision–recall curves for Tier 1 and Tier 2. Supplementary Table S9. Complementary metrics: average precision and Matthews correlation coefficient. Supplementary Table S10. Age-related sensitivity analyses (post hoc). Supplementary Table S11. Lower-teeth-label sensitivity analysis for Tier 2.

## References

[1] Krishan K., Chatterjee P.M., Kanchan T., Kaur S., Baryah N., Singh R. (2016). A review of sex estimation techniques during examination of skeletal remains in forensic anthropology casework. Forensic Sci. Int..

[2] Bewes J., Low A., Morphett A., Pate F.D., Henneberg M. (2019). Artificial intelligence for sex determination of skeletal remains: Application of a deep learning artificial neural network to human skulls. J. Forensic Leg. Med..

[3] Çiftçi R., Dönmez E., Kurtoğlu A., Eken Ö., Samee N.A., Alkanhel R.I. (2024). Human gender estimation from CT images of skull using deep feature selection and feature fusion. Sci. Rep..

[4] Munhoz L., Okada S., Hisatomi M., Yanagi Y., Arita E.S., Asaumi J. (2024). Are computed tomography images of the mandible useful in age and sex determination? A forensic science meta-analysis. J. Forensic Odonto-Stomatol..

[5] Wang X., Liu G., Wu Q., Zheng Y., Song F., Li Y. (2024). Sex estimation techniques based on skulls in forensic anthropology: A scoping review. PLoS ONE.

[6] Toneva D.H., Nikolova S.Y., Fileva N.F., Zlatareva D.K. (2023). Size and shape of human mandible: Sex differences and influence of age on sex estimation accuracy. Leg. Med..

[7] Bertsatos A., Athanasopoulou K., Chovalopoulou M.E. (2019). Estimating sex using discriminant analysis of mandibular measurements from a modern Greek sample. Egypt. J. Forensic Sci..

[8] Saini V., Chowdhry A., Mehta M. (2022). Sexual dimorphism and population variation in mandibular variables: A study on a contemporary Indian population. Anthropol. Sci..

[9] Lin C., Jiao B., Liu S., Guan F., Chung N.E., Han S.H., Lee U.Y. (2014). Sex determination from the mandibular ramus flexure of Koreans by discrimination function analysis using three-dimensional mandible models. Forensic Sci. Int..

[10] Loth S.R., Henneberg M. (1996). Mandibular ramus flexure: A new morphologic indicator of sexual dimorphism in the human skeleton. Am. J. Phys. Anthropol. Off. Publ. Am. Assoc. Phys. Anthropol..

[11] Donnelly S.M., Hens S.M., Rogers N.L., Schneider K.L. (1998). A blind test of mandibular ramus flexure as a morphologic indicator of sexual dimorphism in the human skeleton. Am. J. Phys. Anthropol. Off. Publ. Am. Assoc. Phys. Anthropol..

[12] Hill C.A. (2000). Evaluating mandibular ramus flexure as a morphological indicator of sex. Am. J. Phys. Anthropol. Off. Publ. Am. Assoc. Phys. Anthropol..

[13] Tunis T.S., Sarig R., Cohen H., Medlej B., Peled N., May H. (2017). Sex estimation using computed tomography of the mandible. Int. J. Leg. Med..

[14] Dong H., Deng M., Wang W., Zhang J., Mu J., Zhu G. (2015). Sexual dimorphism of the mandible in a contemporary Chinese Han population. Forensic Sci. Int..

[15] Zheng J., Ni S., Wang Y., Zhang B., Teng Y., Jiang S. (2018). Sex determination of Han adults in Northeast China using cone beam computer tomography. Forensic Sci. Int..

[16] Ceballos F., Deana N., Alves N. (2025). Sex estimation in a Chilean population by mandibular analysis in cone beam computed tomography images. BMC Oral Health.

[17] Christoloukas N., Mitsea A., Kovatsi L., Angelopoulos C. (2025). Sex determination using linear anthropometric measurements relative to the mandibular reference plane on CBCT 3D images. J. Imaging.

[18] Rmoutilová R., Piskačová K., Pilmann Kotěrová A., Dupej J., Bejdová Š., Velemínská J., Brůžek J. (2024). Classification performance of the Sella-Tunis et al. (2017) sex estimation method in Czech population: Different posterior probability threshold approaches. Int. J. Leg. Med..

[19] Chalazoniti A., Lattanzi W., Halazonetis D.J. (2024). Shape variation and sex differences of the adult human mandible evaluated by geometric morphometrics. Sci. Rep..

[20] Godinho R.M., Crevecoeur I., Garcia S., Whiting R., Aramendi J. (2026). Coupling geometric morphometrics and machine learning for mandibular sex estimation in Late Pleistocene and Late Modern populations. Sci. Rep..

[21] Costa Mendes L., Delrieu J., Gillet C., Telmon N., Maret D., Savall F. (2021). Sexual dimorphism of the mandibular conformational changes in aging human adults: A multislice computed tomographic study by geometric morphometrics. PLoS ONE.

[22] Handayani V.W., Margareth Amiatun Ruth M.S., Rulaningtyas R., Caesarardhi M.R., Yudhantorro B.A., Yudianto A. (2025). Development and evaluation of a convolutional neural network model for sex prediction using cephalometric radiographs and cranial photographs. BMC Med. Imaging.

[23] Handayani V.W., Margaretha Amiatun Ruth M.S., Rulaningtyas R., Kurniawan A., Yudhantorro B.A., Yudianto A. (2026). Deep learning for age estimation and sex prediction using mandibular-cropped cephalometric images: Comparative model development and validation study. JMIR AI.

[24] Kuha A., Ackermann J., Junno J.A., Oettlé A., Oura P. (2024). Deep learning in sex estimation from photographed human mandible using the Human Osteological Research Collection. Leg. Med..

[25] Shimkus E., Kružić I., Mladenović S., Perić I., Gunjača M.J., Tadić T., Dolić K., Andelinović Š., Bašić Ž., Jerković I. (2025). Classifying Sex from MSCT-Derived 3D Mandibular Models Using an Adapted PointNet++ Deep Learning Approach in a Croatian Population. J. Imaging.

[26] Secgin Y., Cakmak M., Senol D., Ozturk O., Kaya S., Harmandaoglu O., Biyik E.Y., Yenigül H., Önbaş Ö. (2026). Sex prediction based on computed tomography images of the mandible using deep learning models. Aust. J. Forensic Sci..

[27] Pertek H., Kamaşak M., Kotan S., Hatipoğlu F.P., Hatipoğlu Ö., Köse T.E. (2024). Comparison of mandibular morphometric parameters in digital panoramic radiography in gender determination using machine learning. Oral Radiol..

[28] Corda J.V., Karthikeyan A., Zuber M., Jacob M., Ramos A., Hosapatna M., Dsouza A., Pandey A.K., Ankolekar V.H. (2025). Estimation of sexual dimorphism of adult human mandibles of South Indian origin using non-metric parameters and machine learning classification algorithms. Sci. Rep..

[29] Pishghadam N., Esmaeilyfard R., Paknahad M. (2025). Explainable deep learning for age and gender estimation in dental CBCT scans using attention mechanisms and multi task learning. Sci. Rep..

[30] Noel L., Fat S.C., Causey J.L., Dong W., Stubblefield J., Szymanski K., Chang J.H., Wang P.Z., Moore J.H., Ray E. (2024). Sex classification of 3D skull images using deep neural networks. Sci. Rep..

[31] Han Y., Wang G. (2020). Skeletal bone age prediction based on a deep residual network with spatial transformer. Comput. Methods Programs Biomed..

[32] Su L., Fu X., Hu Q. (2021). Generative adversarial network based data augmentation and gender-last training strategy with application to bone age assessment. Comput. Methods Programs Biomed..

[33] Wasserthal J., Breit H.C., Meyer M.T., Pradella M., Hinck D., Sauter A.W., Heye T., Boll D.T., Cyriac J., Yang S. (2023). TotalSegmentator: Robust segmentation of 104 anatomic structures in CT images. Radiol. Artif. Intell..

[34] Isensee F., Jaeger P.F., Kohl S.A., Petersen J., Maier-Hein K.H. (2021). nnU-Net: A self-configuring method for deep learning-based biomedical image segmentation. Nat. Methods.

[35] Verhelst P.J., Smolders A., Beznik T., Meewis J., Vandemeulebroucke A., Shaheen E., Van Gerven A., Willems H., Politis C., Jacobs R. (2021). Layered deep learning for automatic mandibular segmentation in cone-beam computed tomography. J. Dent..

[36] Ioffe S., Szegedy C. Batch normalization: Accelerating deep network training by reducing internal covariate shift. Proceedings of the International Conference on Machine Learning.

[37] Hu J., Shen L., Sun G. (2018). Squeeze-and-excitation networks. Proceedings of the IEEE Conference on Computer Vision and Pattern Recognition.

[38] He K., Zhang X., Ren S., Sun J. (2016). Deep residual learning for image recognition. Proceedings of the IEEE Conference on Computer Vision and Pattern Recognition.

[39] Loshchilov I., Hutter F. (2017). Decoupled weight decay regularization. arXiv.

[40] Guo C., Pleiss G., Sun Y., Weinberger K.Q. On calibration of modern neural networks. Proceedings of the International Conference on Machine Learning.

[41] Franklin D., Oxnard C.E., O’higgins P., Dadour I. (2007). Sexual dimorphism in the subadult mandible. J. Forensic Sci..

[42] He H., Garcia E.A. (2009). Learning from imbalanced data. IEEE Trans. Knowl. Data Eng..

[43] Brodersen K.H., Ong C.S., Stephan K.E., Buhmann J.M. (2010). The balanced accuracy and its posterior distribution. Proceedings of the 2010 20th International Conference on Pattern Recognition.

[44] McNemar Q. (1947). Note on the sampling error of the difference between correlated proportions or percentages. Psychometrika.

[45] Efron B., Tibshirani R.J. (1993). An introduction to the bootstrap. Monogr. Stat. Appl. Probab..

[46] Selvaraju R.R., Cogswell M., Das A., Vedantam R., Parikh D., Batra D. (2020). Grad-CAM: Visual explanations from deep networks via gradient-based localization. Int. J. Comput. Vis..

[47] Coquerelle M., Bookstein F.L., Braga J., Halazonetis D.J., Weber G.W., Mitteroecker P. (2011). Sexual dimorphism of the human mandible and its association with dental development. Am. J. Phys. Anthropol..

[48] Kotěrová A., Velemínská J., Dupej J., Brzobohatá H., Pilný A., Brůžek J. (2017). Disregarding population specificity: Its influence on the sex assessment methods from the tibia. Int. J. Leg. Med..

