# Supplementary material for: A Tiered 3D CNN Framework for Forensic Sex Estimation from CBCT: Matched Comparison of Full-Skull Volumes and Mandible-Only Masks
Source: Biology (Basel). 2026 Oct 20;15(20):1891. doi: 10.3390/biology15201891 (PMC13701638; doi:10.3390/biology15201891)
Supplement: Supplementary file 1 [file biology-15-01891-s001.zip › biology-4595299-supplementary.pdf]

Supplementary Material

A tiered 3D CNN framework for forensic sex estimation from CBCT: Matched comparison of full-skull volumes and mandible-only masks

This document contains supplementary materials accompanying the main manuscript. Section, Table, Figure, and equation numbers follow the main manuscript unless otherwise stated. Content is organized as follows:

- Supplementary Figure S1. Grad-CAM contact sheet: eight representative Tier 2 correct classifications (Panels a–h).
- Supplementary Figure S2. ROC curves for Tier 1, Tier 2, and the Tier 2 adult subgroup across pooled out-of-fold predictions.
- Supplementary Note S1. Exploratory age-stratified dual-branch fusion analysis and nested rule-selection stress test (archival).
- Supplementary Note S2. Full SE ablation for the Tier 2 mandibular path.
- Supplementary Note S3. ResNet3D-18 contextual reference (architecture comparison with cohort and schedule caveats).
- Supplementary Figure S3. Per-fold balanced accuracy across the tiered framework (Tier 1 / Tier 2 / Tier 2 adult subgroup).
- Supplementary Table S1. Summary of representative mandibular sex estimation studies (cross-study literature comparison).
- Supplementary Table S2. Tier-wise age-stratified and adult-subgroup performance summaries.
- Supplementary Table S3. CBCT acquisition audit summary (scanner configuration and scan-parameter distribution).
- Supplementary Table S4. Hyperparameters of the four morphometric baseline classifiers and the volume-only baseline.
- Supplementary Table S5. Locked age-stratified fusion weights and thresholds (exploratory, archival), (presented within Supplementary Note S1).
- Supplementary Table S6. Tier 2 fixed-window single-branch performance, no-SE versus SE (presented within Supplementary Note S2).
- Supplementary Table S7. Fold composition audit for the seed-42 five-fold split (support for Section 3.4 cross-fold variability and Limitation 3).
- Supplementary Table S8. End-to-end repeated five-fold sensitivity for Tier 2.
- Supplementary Figure S4. Pooled out-of-fold precision–recall curves for Tier 1 and Tier 2.
- Supplementary Table S9. Complementary metrics: average precision and Matthews correlation coefficient.
- Supplementary Table S10. Age-related sensitivity analyses (post hoc).
- Supplementary Table S11. Lower-teeth-label sensitivity analysis for Tier 2.

Supplementary Figure S1. Grad-CAM Contact Sheet: Correct Classifications

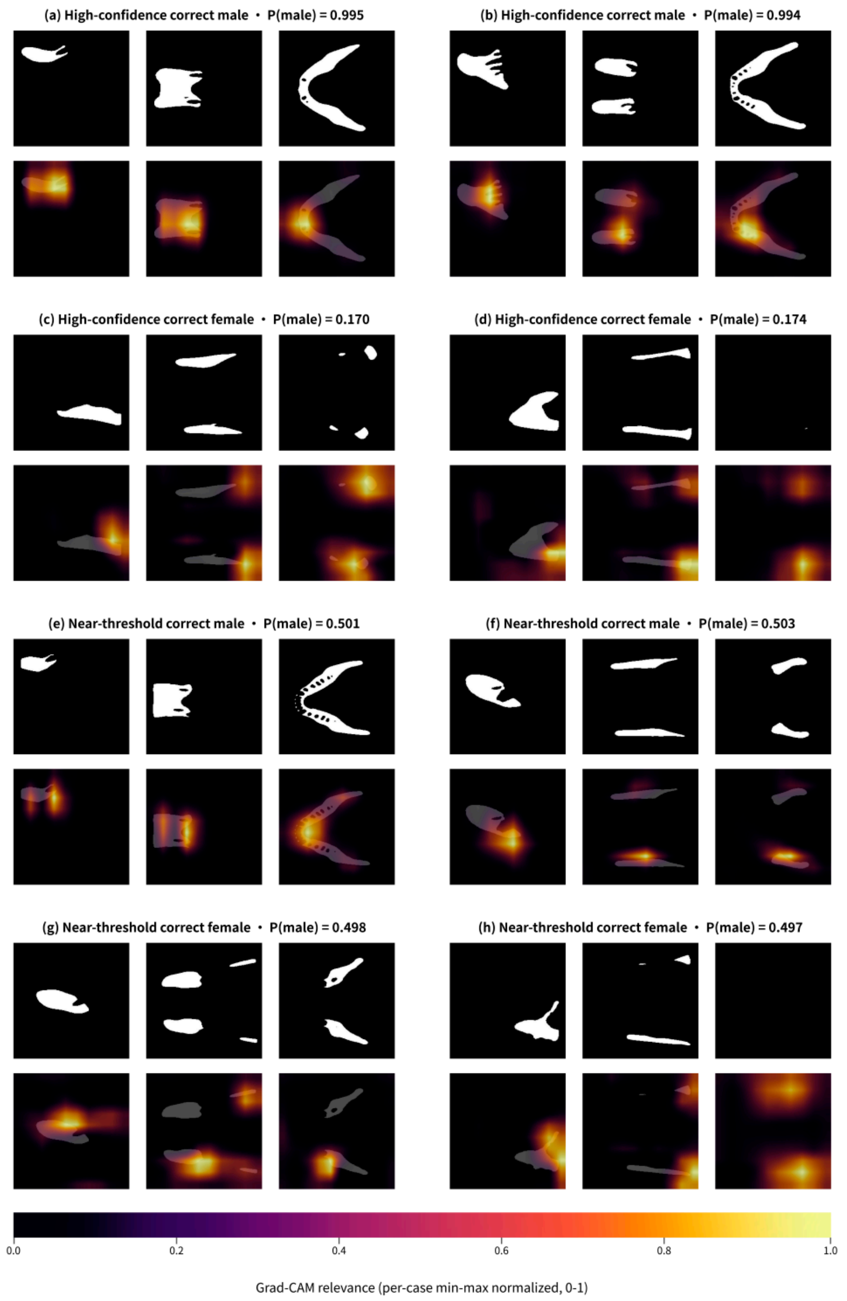

Figure S1. Grad-CAM contact sheet of eight correct Tier 2 classifications from the no-SE mandibular model, spanning high-confidence and near-threshold cases of both sexes. Each panel shows three orthogonal views, with the raw mandibular mask on top and the Grad-CAM overlay below. Maps are min-max normalized within each case, so absolute relevance is not comparable across panels.

Supplementary Figure S2. Pooled Out-of-Fold ROC Curves

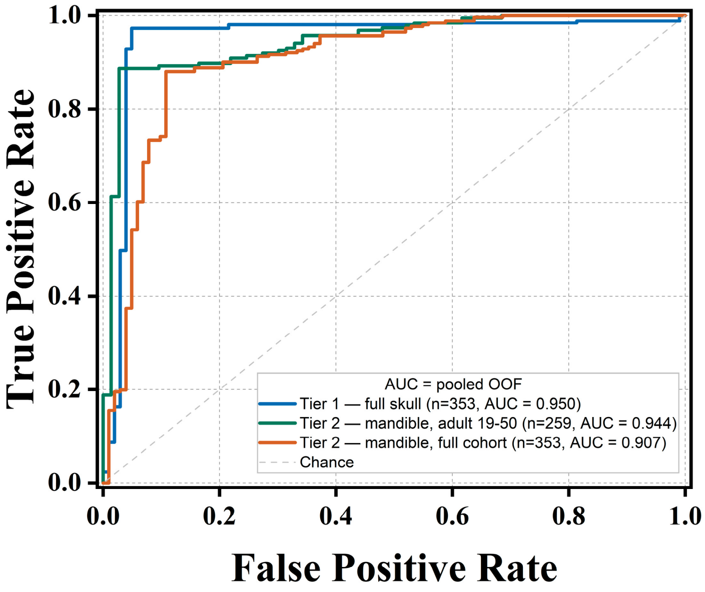

Figure S2. Pooled out-of-fold ROC curves for Tier 1 (full-skull CBCT, n = 353), Tier 2 (mandibular binary mask, n = 353), and the Tier 2 adult subgroup (ages 19–50, n = 259). Female is the positive class. AUC values are computed from a single pooled ROC over all five-fold OOF probabilities.

Supplementary Note S1. Exploratory Age-Stratified Dual-Branch Fusion Analysis

S1.1 Motivation and archival purpose

During early framework development, prior to adopting the tiered design reported in the main manuscript, an alternative approach combining the whole-volume and fixed-window mandibular preprocessing branches through an age-stratified decision-fusion rule was examined. Under the final tiered framework, the whole-volume mandibular branch was not retained as a reported tier: the primary full-skull path (Tier 1) uses complete craniofacial grayscale CBCT input and the secondary mandibular path (Tier 2) uses the fixed-window no-SE variant. This Supplementary Note archives the full numerical characterization of the exploratory fusion analysis for methodological transparency. None of the fusion-derived quantities reported here feed into the Tier 1, Tier 2, or Tier 2 adult subgroup headline metrics of the main manuscript.

S1.2 Fusion formulation

The age-stratified fusion rule combines the male-class probabilities of the whole-volume branch (p_{whole}) and the fixed-window branch (p_{fix}) as a weighted average $p_{fused}=\alpha_k \cdot p_{\mathrm{whole}}+(1-\alpha_k)\cdot p_{fix}$, with age-specific weights α_k and male decision thresholds τ_k for the four age buckets $k\in\{\leq 18, 19–30, 31–50, 51+\}$. Both α_k and τ_k were selected on pooled out-of-fold (OOF) predictions and locked for evaluation reporting.

S1.3 Locked fusion configuration

Table S5

Locked age-stratified fusion weights and thresholds (exploratory, archival).

Age bucket	α_whole	α_fix	Male threshold	Rationale
≤18 years	0.00	1.00	0.516	Developing dimorphism, fixed-window dominates
19–30 years	0.12	0.88	0.507	Mature dimorphism, fixed-window primary
31–50 years	0.00	1.00	0.469	Stable dimorphism, fixed-window dominates
51+ years	0.88	0.12	0.485	Age-related resorption, whole-volume primary

S1.4 Fusion performance under locked rules

Under the locked fusion configuration, fold-mean accuracy was 89.01% ± 2.31% and fold-mean balanced accuracy (BAcc) was 88.75% ± 2.04% (pooled OOF: Acc 89.01%, BAcc 88.78%). Fold-wise BAcc ranged from 86.86% to 91.62%. The fold-level standard deviation (2.04%) was smaller than that of either constituent single branch (whole-volume: 6.13%, fixed-window no-SE: 3.79%), suggesting that the age-stratified rule primarily stabilized performance across folds rather than producing a large absolute accuracy gain. These exploratory single-branch standard deviations predate the plateau-scheduled protocol and therefore differ from the final Tier 2 value of ± 4.58% reported in the main manuscript.

S1.5 Nested rule-selection stress test

Because the fusion weights and thresholds in Table S5 were selected against the same pooled OOF predictions used to score the locked fusion, the locked fusion estimate likely contains a component of post-selection optimism. To quantify this, we performed a nested rule-selection stress test on the frozen branch-level OOF probabilities: for each outer fold, the fusion rules were re-optimized on the remaining four folds' OOF predictions and applied to the held-out fold without further tuning. Base-model retraining within each outer split was not performed. The test operates on frozen OOF probabilities and therefore quantifies rule-selection optimism but not base-model optimism.

Under the nested protocol, fold-mean BAcc decreased to 83.89% ± 8.41% and fold-mean accuracy decreased to 84.23% ± 7.93%, representing a 4.86 percentage-point drop in BAcc relative to the locked configuration. Critically, the nested estimate did not exceed either constituent single branch, indicating that the fusion layer did not provide a robust additive benefit once rule selection was prevented from using the evaluation fold.

S1.6 Why fusion was not retained

Three considerations motivated the transition from the exploratory fusion design to the tiered framework reported in the main manuscript. First, the 4.86 pp nested drop indicated that the apparent locked-fusion advantage was largely post-selection optimism rather than a robust additive gain. Second, introducing an age-conditioned decision layer complicates the forensic-deployment narrative: the Tier 2 mandibular path in the current framework uses no age metadata in inference, and the Tier 2 adult subgroup performance report uses the 19–50 window only for demographic subgrouping, not as a branch-selection rule. Third, under the revised plateau-scheduled training protocol, the Tier 2 fixed-window no-SE model reached BAcc 88.67% ± 4.58% without any fusion, removing most of the practical motivation for the additional complexity.

Supplementary Note S2. Full SE Ablation for the Tier 2 Mandibular Path

S2.1 Motivation

The main manuscript retains the no-SE (no squeeze-and-excitation) variant as the Tier 2 architecture on the grounds that SE-enabled and SE-disabled variants did not differ at a statistically distinguishable level on this cohort. This Supplementary Note documents the fold-mean results of that ablation, paired case-level statistical comparisons, and complementarity-oriented analyses retained from the exploratory fusion work described in Supplementary Note S1.

S2.2 Single-branch performance comparison

Table S6 reports fold-mean accuracy, balanced accuracy, and per-class recall for the Tier 2 fixed-window no-SE and SE-enabled variants under the unified plateau-scheduled training protocol used in the main manuscript. Both variants use identical fold assignments (seed = 42), class-imbalance handling, and optimization settings.

Table S6

Tier 2 fixed-window single-branch performance: no-SE (retained) vs. SE (ablation).

Variant	Fold-mean Acc (%)	Fold-mean BAcc (%)	Male recall (mean)	Female recall (mean)	Params
no-SE (retained)	88.41 ± 5.00	88.67 ± 4.58	0.893 ± 0.053	0.880 ± 0.057	572,338
SE (ablation only)	87.32 ± 7.11	85.84 ± 6.88	0.823 ± 0.075	0.893 ± 0.078	583,218
Δ (no-SE − SE)	+1.09	+2.84	+0.070	−0.013	−10,880

S2.3 Paired case-level statistical comparison

On pooled case-level OOF predictions (n = 353), the paired no-SE vs. SE comparison yielded McNemar exact p = 0.672 (27 discordant cases favoring no-SE, 23 favoring SE). Stratified paired bootstrap with 5,000 resamples, stratified by sex label, gave 95% confidence intervals for the no-SE minus SE difference of [−2.82%, +5.07%] for accuracy, [−2.09%, +5.77%] for balanced accuracy, and [−2.68%, +3.47%] for the female-positive AUC, matching the sign convention of the Table S6 deltas. None of the three confidence intervals excluded zero.

Conclusion: SE did not confer a statistically distinguishable single-branch advantage over the no-SE baseline on this cohort. The no-SE variant was therefore retained as the Tier 2 architecture in the main manuscript, with the additional operational benefit of lower parameter count (572,338 vs. 583,218).

S2.4 Cross-branch complementarity analysis (archival)

The SE ablation was also examined in the context of the exploratory dual-branch fusion analysis described in Supplementary Note S1. Although single-branch SE did not improve accuracy, SE was associated with greater cross-branch prediction diversity when paired with the whole-volume branch under age-stratified fusion:

- Cross-branch prediction disagreement (pooled OOF): 14.6% for the SE pairing vs. 12.4% for the no-SE pairing.
- Both-branches-incorrect count: 25 cases for SE pairing vs. 31 cases for no-SE pairing.
- Branch-level oracle BAcc (upper bound when either branch is correct): 91.84% for SE pairing vs. 91.24% for no-SE pairing.
- Age-stratified fusion weights: SE pairing retained non-zero whole-volume contributions in two of four age buckets (19–30: α_whole = 0.12, 51+: α_whole = 0.88), whereas the no-SE pairing collapsed to α_whole = 0.00 in all four buckets, effectively reducing to a single-branch model with age-conditioned thresholds.

These observations are consistent with the interpretation that SE, while not improving aggregate single-branch accuracy, shifted the model's error profile sufficiently to enable modest cross-branch complementarity under fusion. As established in Supplementary Note S1, the fusion benefit itself was not robust under nested rule selection. This complementarity is reported as exploratory only.

S2.5 Summary

Under the internal evaluation protocol used in the main manuscript (five-fold stratified cross-validation with matched fold assignments, class-weighted cross-entropy with weighted random sampling, plateau-scheduled AdamW training, and case-level paired statistical testing), squeeze-and-excitation channel attention did not confer a statistically distinguishable single-branch advantage for Tier 2 mandibular sex classification on this cohort. The no-SE variant was retained on grounds of comparable accuracy, lower parameter count, and marginally lower fold-level variance. The complementarity observations reported in S2.4 pertain to an exploratory fusion configuration that is not part of the main tiered framework.

S2.6 Grad-CAM reproducibility check

The Grad-CAM visualizations shown in main manuscript Fig. 4 and Supplementary Figure S1 were generated on the archived final no-SE Tier 2 checkpoints used for the main-manuscript results. The Grad-CAM rerun reproduced the saved out-of-fold logits to numerical precision (mean $|\Delta \text{pmale}| = 2.53 \times 10^{-8}$, with the final convolutional block as the target layer), confirming that the attention maps are derived from the exact model instance used for the main-manuscript metrics rather than from a re-initialized or re-trained variant.

Supplementary Note S3. ResNet3D-18 Contextual Reference

S3.1 Motivation

A larger 3D CNN architecture (ResNet3D-18 [38]) was evaluated as a contextual reference to position the proposed lightweight backbone within the deep-learning baselines literature. Two design differences relative to the main-manuscript Tier 1 / Tier 2 pipeline preclude direct head-to-head ranking and motivate reporting this comparison as a Supplementary contextual reference rather than a Tier-level result.

S3.2 Implementation

The model was implemented as a custom single-channel adaptation of torchvision.models.video.r3d_18 (weights = None, ~33 M trainable parameters). The stem conv1 was replaced by a single-channel 3D convolution to accept the 80^3 mandibular binary-mask input. The model was trained from scratch under the Tier 2 preprocessing pipeline, AdamW optimizer (initial learning rate 5×10^{-4}, weight decay 1×10^{-3}), 5-epoch linear warmup followed by cosine annealing with T_{max} = 95 epochs, label smoothing 0.1, effective batch size 16, and early stopping on validation BAcc (patience = 20). It was evaluated under five-fold stratified cross-validation on the same protocol structure as the proposed lightweight backbone.

S3.3 Performance and design caveats

Under five-fold stratified cross-validation, ResNet3D-18 achieved a mean balanced accuracy of 84.84% ± 6.27%, below the proposed lightweight Tier 2 backbone (88.67% ± 4.58%) on the matched mandibular input. Two design differences should be noted. First, the ResNet3D-18 evaluation was conducted on an earlier cohort version prior to the final quality-control exclusions (final analytic cohort N = 353), and was not re-run on the final N = 353 fold assignment. The comparison is therefore contextual rather than strictly cohort-matched. Second, the optimizer schedule (cosine annealing) was not matched to the plateau-scheduled protocol used for the Tier 1 / Tier 2 lightweight backbone, and the result therefore should not be interpreted as a fully schedule-controlled architecture ablation. These caveats are noted to bound the interpretation of the ResNet3D-18 result. The main-manuscript Tier 1 / Tier 2 comparison and statistical inference (Section 3.5, paired bootstrap and McNemar tests) operate exclusively on the matched N = 353 cohort and are unaffected by these caveats.

Supplementary Figure S3. Per-Fold Balanced Accuracy Across the Tiered Framework

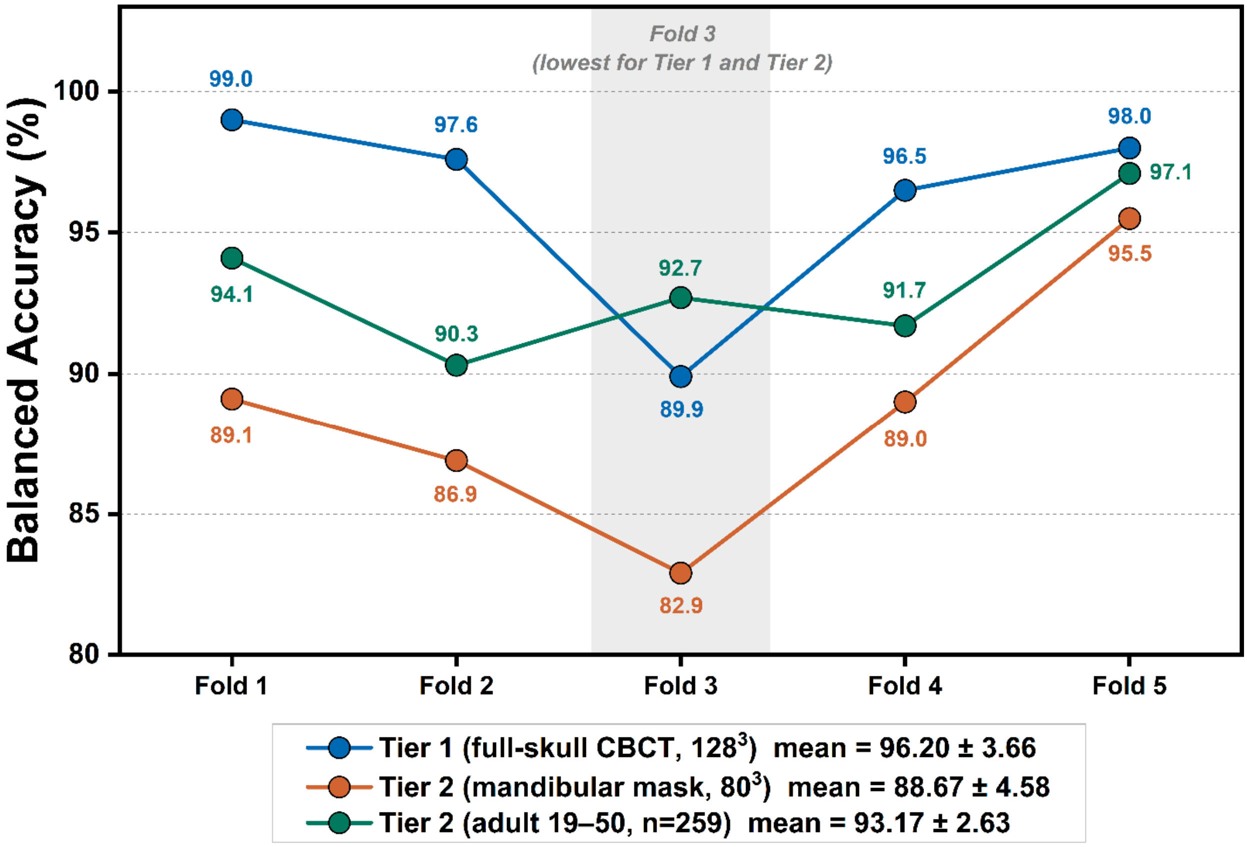

Figure S3. Per-fold balanced accuracy across the tiered framework under the seed = 42 stratified five-fold cross-validation. Each line tracks one configuration across the five folds. The three configurations are Tier 1 (full skull, n = 353), Tier 2 (mandibular mask, n = 353, no-SE), and the Tier 2 adult subgroup (ages 19–50, n = 259). Fold-mean BAcc values of 96.20%, 88.67%, and 93.17% appear in the legend. Fold 3 is the lowest-performing split for Tier 1 and Tier 2.

Supplementary Table S1. Cross-Study Literature Comparison

Table S1 summarizes representative prior work on mandibular (and closely related craniofacial) sex estimation, organized by methodological family. The present study is listed at the bottom for direct comparison. Accuracy values are reported as in the original source. Fold-mean ± SD is given where available, and single-point values are labeled as reported.

Study	Population / cohort	Input / method	Reported performance (metric as reported in source)	Metric type	Ref
Bertsatos et al. 2019	Greek mandibles	DFA, 20 linear + 3 angular meas.	85.7% (cross-validated)	Accuracy	[7]
Saini et al. 2022	North Indian mandibles	DFA, 17 variables	84.7%	Accuracy	[8]
Lin et al. 2014	Korean mandibles	DFA, 11 3D measurements	88.8%	Accuracy	[9]
Sella-Tunis et al. 2017	Israeli CT (n = 438)	25 measurements	90.8% (CV 89.0%)	Accuracy	[13]
Dong et al. 2015	Chinese Han CT	Linear measurements	84.2%	Accuracy	[14]
Zheng et al. 2018	Chinese Han CBCT	Linear measurements	87.4%	Accuracy	[15]
Ceballos et al. 2025	Chilean CBCT	Linear measurements	87.1%	Accuracy	[16]
Rmoutilova et al. 2024	Czech (Sella-Tunis method applied)	Transferability test	78–82% (cross-pop.)	Accuracy	[18]
Chalazoniti et al. 2024	Adult mandibles (519 semi-landmarks)	Geometric morphometrics	91%	Accuracy	[19]
Toneva et al. 2023	Adult mandibles	Size vs. shape decomposition	Size 87%, shape 78%, GPA-shape 53%	Accuracy	[6]
Godinho et al. 2026	Portuguese (modern)	GMM + ML, cross-temporal test	~90% in-sample, 60–63% cross-temp.	Accuracy	[20]
Pertek et al. 2024	Turkish panoramic	Naive Bayes	84.0%	Accuracy	[27]
Corda et al. 2025	South Indian mandibles	Random Forest + SMOTE	92%	Accuracy	[28]
Kuha et al. 2024	South African photographed mandibles	2D CNN	Overall ~88%, male 100% / female 76.9%	Accuracy + per-class recall	[24]
Handayani et al. 2025 / 2026	Indonesian cephalometric radiographs	2D CNN, mandibular ROI crop	Male F1 93% → 63% after crop	F1 (male)	[22,23]
Shimkus et al. 2025	Croatian MSCT mandibles (n = 254, sex-balanced)	PointNet++ on 3D point clouds	93% CV, 92% independent test	Accuracy	[25]

Study	Population / cohort	Input / method	Reported performance (metric as reported in source)	Metric type	Ref
Secgin & Cakmak 2026	Turkish mandibular CT	Hybrid deep-learning model on mandibular CT images	92.5%	Accuracy	[26]
Bewes et al. 2019	Australian skull CT	GoogLeNet (2D slice-based)	95%	Accuracy	[2]
Noel et al. 2024	Skull 3D meshes	ResNet3D / PointNet / MeshNet	AUC > 0.9 (3-way comparison)	AUC	[30]
Pishghadam et al. 2025	Iranian CBCT slices	2D CNN + CBAM, multi-task	95.3%	Accuracy	[29]
Present study, Tier 1	Chinese Han CBCT (n = 353, 102 M / 251 F, age 6–78)	Full-skull 3D CNN (~572K params), 128^3 grayscale	96.60% ± 2.97% (BAcc 96.20% ± 3.66%)	Accuracy + BAcc	—
Present study, Tier 2	Chinese Han (same cohort)	Mandibular 3D CNN, 80^3 binary mask	88.41% ± 5.00% (BAcc 88.67% ± 4.58%)	Accuracy + BAcc	—
Present study, Tier 2 adult subgroup	Adult subgroup (19–50, n = 259)	Tier 2 adult subgroup report (same model as Tier 2, not independently trained)	91.12% ± 2.71% (BAcc 93.17% ± 2.63%)	Accuracy + BAcc	—

Note. Cohort sizes, age composition, population, and evaluation protocols differ substantially across the cited studies. The cross-study comparison is therefore intended as contextual rather than a controlled benchmark. Direct numerical comparison between studies should be interpreted cautiously in light of these design differences (cohort composition, age range, sex balance, cross-validation protocol, preprocessing modality).

Supplementary Table S2. Tier-wise age-stratified and adult-subgroup performance summaries

Table S2 reports two complementary summaries. Panel A reports pooled out-of-fold balanced accuracy (BAcc) within each pre-defined age stratum. Panel B reports fold-mean ± SD BAcc for the pre-specified 19–50 adult subgroup, matching the main-text subgroup comparison in Section 3.2.

Panel A

Pooled out-of-fold age-stratum performance.

Age stratum	n (M / F)	Tier 1 BAcc	Tier 2 BAcc	Tier 1 − Tier 2 (pp)
Pediatric ≤17	17 (6 / 11)	75.00%	66.67%	+8.33
Transitional age 18	27 (4 / 23)	100.00%	80.98%	+19.02
Young adult 19–29	206 (50 / 156)	96.08%	93.23%	+2.85
Middle adult 30–50	53 (23 / 30)	100.00%	92.83%	+7.17
Elderly >50	50 (19 / 31)	98.39%	79.80%	+18.59
All ages ≥18 (non-pediatric)	336 (96 / 240)	97.50%	—	—
Full cohort	353 (102 / 251)	96.15%	88.63%	+7.52

Panel B

Fold-mean adult subgroup comparison.

Subgroup	n (M / F)	Estimate type	Tier 1 BAcc	Tier 2 BAcc	Tier 1 − Tier 2 (pp)
Adult 19–50	259 (73/186)	Fold-mean ± SD across five folds	97.12% ± 2.89%	93.17% ± 2.63%	+3.95

**Note:* Panel A values are pooled out-of-fold BAcc values within each stratum. In Panel B, the ± values denote the standard deviation across the five folds. Panel B is included to provide traceability for the adult-subgroup comparison reported in the main text. Tier 1 − Tier 2 differences are descriptive; no stratum-level hypothesis test was applied.

Supplementary Table S3. CBCT Acquisition Audit

Table S3 summarizes the image acquisition characteristics of the 353-case analytic cohort used for Tier 1 (full-skull CBCT) and Tier 2 (mandibular binary mask derived from the same CBCT volumes). All cases were acquired on a single dental/maxillofacial cone-beam CT scanner. This audit was performed retrospectively from DICOM header metadata on the final 353 cases after the quality-control exclusions described in main manuscript Section 2.1.2.

Acquisition parameter	Value
Scanner modality	Dental/maxillofacial cone-beam CT (CBCT)
Manufacturer and device model	LargeV Smart3DX CBCT system (LargeV Instrument Corp. Ltd., Beijing, China)
Number of scanners	1 (single-device cohort)
Tube voltage	100 kVp (uniform across all 353 cases)
Voxel spacing	Isotropic, 0.250 mm for the majority of cases, with two subsidiary isotropic settings (0.088 mm and 0.125 mm) on a small subset
Voxel isotropy	Fully isotropic for all 353 cases
DICOM modality tag	Predominantly labeled CT, with a minor subset labeled DX. Device audit confirms all scans were acquired on the same CBCT scanner regardless of vendor-reported modality tag
Intensity calibration	Vendor-reported nominal intensity scale, not calibrated to true Hounsfield units on the medical-CT quantitative scale

**Note:* Acquisition parameters were extracted from DICOM headers for each of the 353 analytic cases. The vendor-reported modality tag (CT vs. DX) reflects DICOM-field convention rather than the physical imaging technology. The single-device audit confirmed that all cases were produced by the same CBCT scanner.

Supplementary Table S4. Hyperparameters of the four morphometric baseline classifiers and the volume-only baseline.

Table S4 lists the implementation and key hyperparameters of the four morphometric machine-learning baselines and the volume-only baseline described in Section 2.6. The four morphometric classifiers used the same seven shape descriptors. The volume-only baseline used a single feature (total mandibular volume). All baselines used the same five-fold stratified cross-validation (seed 42) on the 353-case cohort as the deep-learning models, with features standardized within each training fold.

Classifier	Implementation	Key hyperparameters
Logistic regression	scikit-learn	L2 penalty, lbfgs solver, C = 1.0, class_weight = 'balanced'
Random forest	scikit-learn	500 trees, Gini impurity, min_samples_leaf = 2, max_features = 'sqrt', class_weight = 'balanced_subsample'
Support vector machine	scikit-learn	RBF kernel, C = 1.0, gamma = 'scale', class_weight = 'balanced'
Multilayer perceptron	PyTorch	Two hidden layers (7 → 64 → 32 → 2), BatchNorm and ReLU, dropout regularization, class-weighted cross-entropy
Volume-only logistic regression	scikit-learn	L2 penalty, lbfgs solver, C = 1.0, class_weight = 'balanced'; single input feature (total mandibular volume, mm^3)

Supplementary Table S7. Fold composition audit for the seed-42 five-fold split.

Table S7 reports the composition of each fold under the seed-42 stratified five-fold split used for both tiers. Age strata follow the definitions in main manuscript Table 4 (pediatric ≤17, transitional age 18, young adult 19–29, middle adult 30–50, elderly >50). This audit supports the interpretation in Section 3.4 and Limitation 3 that the lower Fold 3 performance was not explained by demographic imbalance.

Fold	n	M/F	Median age	Pediatric	Transitional	Young adult	Middle adult	Elderly
1	71	20/51	23	4	6	37	12	12
2	71	21/50	22	4	7	45	9	6
3	71	21/50	23	4	4	44	11	8
4	70	20/50	22	2	4	45	9	10
5	70	20/50	23	3	6	35	12	14

Supplementary Table S8. End-to-end repeated five-fold sensitivity for Tier 2.

Table S8 reports Tier 2 balanced accuracy under three complete five-fold retraining and evaluation runs, each using a different CV/model seed setting. Seed 42 corresponds to the primary analysis.

CV/model seed	Fold-mean BAcc	Pooled OOF BAcc
42	88.67% ± 4.58%	88.63%
2024	88.27% ± 2.07%	88.33%
2025	86.04% ± 3.94%	86.09%

Note: Each row represents a complete five-fold retraining and out-of-fold evaluation under the corresponding CV/model seed setting, in which both the fold assignment and the model training randomness were varied. Seed 42 corresponds to the primary analysis.

Supplementary Figure S4. Pooled Out-of-Fold Precision–Recall Curves

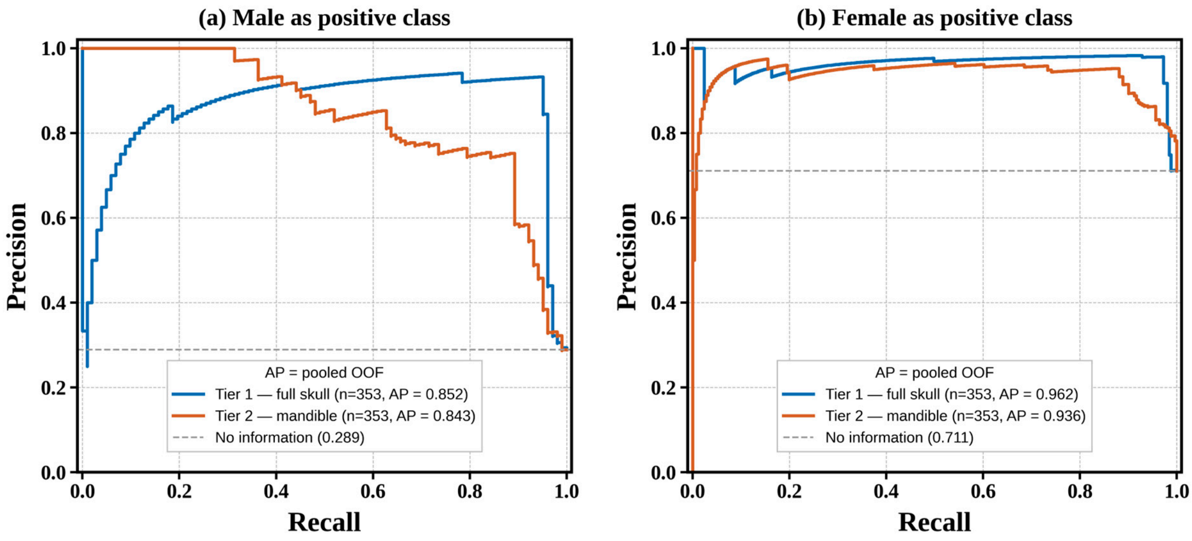

Figure S4. Pooled out-of-fold precision–recall curves for Tier 1 and Tier 2, (a) males as the positive class, (b) females as the positive class; dashed lines mark the no-information precision, and AP values appear in the legend.

Supplementary Table S9. Complementary metrics: average precision and Matthews correlation coefficient.

Metric	Tier 1, fold mean ± SD	Tier 1, pooled	Tier 2, fold mean ± SD	Tier 2, pooled	Paired difference, pooled (95% CI)
MCC	0.919 ± 0.070	0.918	0.742 ± 0.102	0.738	0.180 (0.104 to 0.261)
AP, male positive	0.887 ± 0.114	0.852	0.854 ± 0.077	0.843	0.008 (−0.085 to 0.117)
AP, female positive	0.965 ± 0.047	0.962	0.947 ± 0.039	0.936	0.026 (−0.004 to 0.050)

Note: AP is the step-wise (non-interpolated) area under the precision–recall curve (scikit-learn average_precision_score), computed from the raw out-of-fold scores; no-information precision is 0.289 with males and 0.711 with females as the positive class. Differences are Tier 1 minus Tier 2; 95% CIs are percentile intervals from 5,000 sex-stratified paired bootstrap resamples (cases in identifier order, seed 42).

Supplementary Table S10. Age-related sensitivity analyses (post hoc).

Analysis	Estimate (95% CI)
GEE, misclassification: Tier 2 vs. Tier 1	OR 3.78 (2.02–7.09)
GEE: age ≤18 vs. 19–50 years	OR 2.31 (0.94–5.65)
GEE: age >50 vs. 19–50 years	OR 2.01 (0.98–4.12)
GEE: female vs. male	OR 0.96 (0.46–1.98)
GEE: tier × age-group interaction	Wald χ^2 = 1.49, df = 2, p = 0.47
Tier 2 logistic, sex-adjusted: age ≤18 vs. 19–50 years	OR 2.26 (0.94–5.45), p = 0.069
Tier 2 logistic, sex-adjusted: age >50 vs. 19–50 years	OR 2.61 (1.15–5.91), p = 0.022
Tier 1 logistic, sex-adjusted: age ≤18 vs. 19–50 years	OR 2.40 (0.61–9.49), p = 0.21
Tier 1 logistic, sex-adjusted: age >50 vs. 19–50 years	OR 0.60 (0.07–4.91), p = 0.63
Age-only classifier, pooled BAcc	58.13% (male recall 42.2%, female recall 74.1%)
Age-standardized BAcc, Tier 1 / Tier 2	96.28% / 89.05%

Note: GEE with binomial family, logit link, exchangeable working correlation within cases and robust standard errors (706 observations, 353 clusters); the ≤18 group combines the ≤17 and 18-year strata. Age-standardized BAcc weights male and female recall in the five strata by the whole-cohort stratum proportions.

Supplementary Table S11. Lower-teeth-label sensitivity analysis for Tier 2.

Measure	Original input (mandible label)	Lower-teeth label added	Difference, added minus original (95% CI)
Fold-mean BAcc ± SD (%)	88.67 ± 4.58	86.92 ± 5.21	—
Pooled BAcc (%)	88.63	86.79	−1.84 (−5.77 to 1.79)
Pooled male recall	91/102 (89.22%)	84/102 (82.35%)	—
Pooled female recall	221/251 (88.05%)	229/251 (91.24%)	—
Misclassified cases (male / female)	41 (11 / 30)	40 (18 / 22)	—

Note: Both inputs retain the alveolar process and tooth sockets. The added input also contains the corresponding automatically segmented lower-teeth label. The label was added within each case's original mandibular crop before the original resampling and normalization. In two cases, 0.02% and 0.12% of the label voxels lay outside the crop. Tier 2 was retrained once on the added input with the original case-level fold assignments, seeds, and training protocol. The original input uses the saved predictions of the main analysis. Fold-mean values are means ± sample SD over the five folds. Pooled values use all 353 out-of-fold predictions. The difference is in pooled BAcc (percentage points). Its 95% CI is a percentile interval from 5,000 sex-stratified case-paired bootstrap resamples (cases in identifier order, seed 42). The interval does not include variation between training runs. For paired correctness, 293 cases were correct with both inputs, 21 were incorrect with both, 19 were correct only with the original input, and 20 were correct only with the lower-teeth label. The exact McNemar test ($p = 1.00$) compares overall misclassification. It is not a test of BAcc or of equivalence.
